# Education and active labour market policy complementarities in promoting employment: Reinforcement, substitution and compensation

**DOI:** 10.1111/spol.12894

**Published:** 2023-01-10

**Authors:** Ilze Plavgo

**Affiliations:** ^1^ Department of Political and Social Sciences European University Institute Fiesole Italy

**Keywords:** ALMP, compensation, education, employment, EU‐SILC, institutional complementarities, reinforcement, substitution

## Abstract

This paper theorises and empirically assesses how education and active labour market policy (ALMP) relate to each other in shaping individuals' employment chances in Europe. It provides a theoretical base for assessing policy complementarities building on sociological skill‐formation literature, varieties of capitalism and social investment literature. Two hypotheses of complementarity are advanced: reinforcement whereby higher investment in general skills via education boosts ALMP effectiveness; and substitution‐compensation whereby investments in either policy suffice, rendering individual employment chances less dependent on ALMPs at higher (prior) educational investment levels. The advanced theoretical propositions are empirically tested by looking at how individual employment chances are affected by national ALMP efforts conditional on workforce education, distinguishing between individual‐ and national‐level educational attainment. Analyses draw on micro‐level EU‐SILC longitudinal data 2003–2015 from 29 European countries and 285 country‐years applying mixed‐effects dynamic panel regression models. Results highlight the complementarity of education in the functioning of ALMPs and show that the education‐ALMP interplay follows different dynamics when individual or national education are considered, with substitution‐compensation for the former and reinforcement for the latter. Higher individual educational attainment is associated with lower marginal returns from national ALMP efforts, with higher ALMP effectiveness among the lower‐educated. By contrast, higher national educational attainment is associated with increased ALMP effectiveness, with ALMPs tending to be far less effective at low levels of highly educated workforce. Different interaction patterns are observed for youth, indicating increased difficulty in activating this risk group.

## 1 INTRODUCTION

Welfare state and political economy scholarship increasingly acknowledge the complementarity of education in the functioning of other social policies. The contemporary welfare state approach to dealing with economic and social challenges is geared towards human capital formation, preservation and activation (Garritzmann et al., 2022; Hemerijck, 2017; Morel et al., 2012). This brings education to the core of the welfare state, together with active labour market policies (ALMPs) that facilitate human capital capacitation and labour market (re)insertion. How education and ALMPs interact in affecting individuals' employment chances is therefore an important but currently understudied topic in social sciences.

Recent welfare state scholarship calls for more attention to policy interactions in social policy research (Hemerijck et al., 2022; Yerkes et al., 2022). Interest in policy complementarities in shaping welfare outcomes is not new. Political economy scholars have extensively theorised and assessed how institutional complementarities across sub‐spheres of the macroeconomy shape competitive advantage in market economies, most notably among the ‘varieties of capitalism’ literature (Hall & Gingerich, 2009; Hall & Soskice, 2001; Iversen & Stephens, 2008). Welfare state scholars have for several decades underlined the complementary nature of capacitating and compensatory policies in addressing the new social risks of contemporary knowledge economies (Esping‐Andersen et al., 2002; Hemerijck, 2017).

The empirical assessment of policy complementarities in shaping employment outcomes has become a focus of some of the more recent scholarship (Bakker & Van Vliet, 2021; Hemerijck et al., 2016; Nieuwenhuis, 2022), albeit mainly focusing on macro‐level employment outcomes (exception: Hemerijck et al., 2016). How education and ALMPs interact in affecting employment chances at an individual level is limited to ALMP impact assessments capturing participants of different skill levels with an aim to identify heterogeneity in programme access and effectiveness to uncover potential Matthew Effects (Bonoli & Liechti, 2018). Further theory building and empirical research are necessary to understand the complementary nature of education and labour market policies in shaping people's employment opportunities.

This article follows up on the call to study policy complementarities, focusing on the education‐ALMP interplay. The contribution of this article is both theoretical and empirical. Theoretically, it provides an analytical base for assessing the education‐ALMP interplay, building on the skill formation literature at a micro level (Cunha & Heckman, 2007; DiPrete & Eirich, 2006), social investment scholarship at a policy level (Hemerijck, 2017), and the varieties of capitalism perspective at a state level (Hall & Soskice, 2001; Iversen & Stephens, 2008). Two alternative hypothesized mechanisms of policy complementarity are put forth. First, *reinforcement* whereby higher investments in one policy boost the effectiveness of the other. Following this hypothesis, higher workforce education is expected to lead to higher returns from more ALMP efforts. Second, *substitution‐compensation* whereby higher investments in one policy diminish the effectiveness of the other. Following this hypothesis, higher workforce education leads to diminishing marginal returns to higher ALMP efforts, while lower (prior) educational investments increase ALMP effectiveness. The theoretical framework distinguishes between the moderating role of individual and national level educational attainment in the functioning of ALMPs.

These theoretical propositions are then tested empirically, drawing on micro‐level panel data from 285 longitudinal EU‐SILC surveys from 29 European countries collected between 2003 and 2015 and combined with aggregate data. Analyses assess how individual and national educational attainment interact with national ALMP efforts in promoting employment, for the total workforce and by age group to account for potential heterogeneity across at‐risk groups.

The next section discusses existing research approaches to assessing social policies and their interplay in affecting employment. Section 3 theorises two alternative education‐ALMP complementarity dynamics. Section 4 describes the data and research strategy for testing the hypothesized dynamics. Results in Section 5 show that education and ALMP have strong complementarities in affecting employment probability among individuals living in Europe. The identified interaction dynamics go in opposite directions depending on whether individual or national educational attainment is considered. This has relevant policy implications discussed in the conclusions.

## 2 ASSESSING SOCIAL POLICY WELL‐BEING RETURNS THROUGH THE LENS OF POLICY COMPLEMENTARITIES

Two parallel traditions of welfare state research have dominated the way social policy effects are assessed. On the one hand, social policies are analysed independently from one another, eliminating as much context as possible (e.g., Card et al., 2018). While this approach is useful for isolating the causal effect of one particular policy, it has been repeatedly recognised that welfare provision operates in an interconnected way and requires mutual consistency to operate effectively (Atkinson, 2010; Hemerijck & Plavgo, 2021; Vandenbroucke & Vleminckx, 2011). On the other hand, following the seminal work of *Three Worlds of Welfare Capitalism* by Esping‐Andersen (1990), welfare provisions are studied through the lens of welfare regimes emphasising policy interdependencies and path dependencies within them. Also the varieties of capitalism approach distinguishes between different welfare production regimes, grouping countries into distinct systems (Hall & Soskice, 2001). While regime literature is helpful for understanding welfare state variation, it falls short in grasping within‐regime varieties of policy mixes and over‐time convergence in policy efforts, as indicated by recent empirical research (Ferragina, 2022; Ronchi, 2018).

The potential effects of balancing between social policies are captured by the social investment life course multiplier theorised by Hemerijck (2017). This multiplier logic conceptualises the welfare state as a bundle of interdependent policy provisions that can have reinforcing or weakening effects depending on the presence or absence of certain policy combinations at different moments of people's lives. In its current formulation, the life course multiplier concept offers a basis for theoretical propositions but lacks theorization on the mechanisms by which policy bundles generate different socioeconomic outcomes. Nonetheless, as we shall see below, the multiplier concept is helpful for theorising the education‐ALMP interplay as it foresees complementarity between stock‐enhancing and activating policies in producing welfare outcomes, both at an individual and national level.

Education and ALMP effectiveness in promoting employment have been studied extensively in policy research, albeit mostly independently from each other. Within this unidimensional strand of research, investments in ALMPs and education have mostly been found to positively affect employment chances and income (Bradley & Stephens, 2007; Card et al., 2018; Hanushek et al., 2017; Rovny, 2014; Taylor‐Gooby et al., 2015). It has also been noted that such investments are distributed unequally, and their effects may vary depending on individual characteristics. Some scholars argue that general education and ALMPs positively affect employment chances of disadvantaged groups such as youth (Hanushek et al., 2017). Others underline that in certain contexts, policies targeted at upskilling and activation may have Matthew Effects as they can promote employment for labour market ‘insiders’ at the expense of harder‐to‐place risk groups (Bonoli et al., 2017; Bonoli & Liechti, 2018; Van Lancker & Ghysels, 2012).

How policies targeted at upskilling and activation relate to each other is an understudied but gradually growing field of enquiry. Empirically, Hemerijck et al. (2016) examined the joint effects of ALMPs and early childhood education and care policies (ECEC) on individuals' employment probability and found that ALMP spending has a stronger positive association with employment for certain risk groups when investments in ECEC services are higher. Bakker and Van Vliet (2021) and Nieuwenhuis (2022) studied the interplay of the same two policies in affecting aggregate‐level employment rates and found a substitution effect with diminishing marginal returns to further investments in one policy when investments in the other are high. These studies are highly valuable in improving our understanding about policy interactions and point at different theoretical insights from micro and macro‐level analyses.

Comparative research on how national workforce education and ALMPs relate with each other in affecting employment is scarce, with available studies using public spending indicators and finding no interaction effects (Bakker & Van Vliet, 2021). Research on how individual‐level education and ALMPs relate to each other has received far more attention, predominantly in programme‐specific ALMP impact evaluation studies. A systematic review of 87 ALMP evaluation studies by Bonoli and Liechti (2018) shows that overall ALMPs tend to have a positive access bias benefitting lower‐educated participants more, although in some countries and for certain ALMP programmes, the highly educated were found to gain more than the lower educated from ALMP provision. According to the authors, the observed cross‐country variation in ALMP access bias may be linked to country differences in the general cognitive skill level of the adult population (Bonoli & Liechti, 2018, p. 908). This underlines the importance of considering education not only at an individual but also national level to better understand the education‐ALMP interplay.

## 3 THEORISING POLICY COMPLEMENTARITIES: REINFORCEMENT, SUBSTITUTION AND COMPENSATION

The varieties of capitalism literature has prominently advanced the notion of institutional complementarities, highlighting different dynamics by which institutions interact in producing welfare outcomes. The first and the most widely emphasised is the complementarity dynamic whereby ‘Two institutions can be said to be complementary if the presence (or efficiency) of one increases the returns from (or efficiency of) the other’ (Hall & Soskice, 2001, p. 17). The second, receiving less attention among this scholarship, is the substitution dynamic whereby ‘Two institutions can be said to be “substitutable” if the absence or inefficiency of one increases the returns to using the other’ (Hall & Soskice, 2001, p. 17).

This conceptualization has also been used in social policy research to describe how policies relate to each other in producing social outcomes. Policies are seen as mutually complementary or reinforcing when investment in any one policy facilitates the effectiveness of the other (Hemerijck, 2017; Hemerijck et al., 2016; Nieuwenhuis, 2022). By contrast, policies are mutually dampening or substitutionary when the presence of one reduces the effectiveness of the other, or when the presence of one leads to diminishing marginal returns from investing in the other (Bakker & Van Vliet, 2021; Nieuwenhuis, 2022). Substitution can take place when policies serve a similar purpose and when having either policy is sufficient to reach that purpose (Nieuwenhuis, 2022, p. 6). Inferring from the substitution dynamics, the absence of either one policy serving a similar purpose can be compensated by the presence of the other.

While the concept of institutional complementarities concerns primarily macroeconomic welfare outcomes, policies affect people's lives at an individual level. The social investment life‐course multiplier introduced by Hemerijck (2017) aims at capturing both the macro‐ and micro‐ level dynamics of policy interactions. It posits that well‐being returns to social investments are cumulatively reinforced by the presence of complementary policy combinations or ‘bundles’ that people are exposed to over their life course (Hemerijck et al., 2022, p. 8). How exactly these policy bundles cumulatively reinforce each other and whether complementarity is expected to follow different dynamics at a micro and macro level is not explicitly described and requires further theorization. The rest of the section delves into the theoretical mechanisms explaining the education‐ALMP interplay, distinguishing between reinforcement, substitutability and compensation.

### 3.1 Reinforcement

There are good reasons to believe that educational investments not only positively affect employment outcomes, but also reinforce returns to investment in active labour market policies. A dominant explanation for investment complementarity is anchored in the cumulative advantage theory from social stratification research (DiPrete & Eirich, 2006) and human capital formation theory (Becker, 1993; Cunha & Heckman, 2007). According to these literature strands, skills acquisition is a cumulative, hierarchical process, dynamically produced starting from childhood and accumulated over time. Path‐dependency in skill formation implies that the current skill level is a positive function of previously acquired skills. Stocks of skills acquired in the initial period have a reinforcing effect as they make investments in the subsequent period more productive.

Based on this skills‐beget‐skills mechanism, we can expect that the higher the workforce educational attainment the higher the returns from ALMP programmes. This can stem from both higher likelihood to be enrolled and higher ALMP effectiveness once enrolled. A lower educated workforce may face access bias, with more effective ALMPs reserved to those considered as promising in terms of labour market re‐entry due to labour market selectivity and cream‐skimming by ALMP institutions (Bonoli & Liechti, 2018). Moreover, many of the training and activation interventions require pre‐existing cognitive skills. Since early interventions tend to have higher returns than later interventions (Heckman, 2006, pp. 1901–1902), it may be less effective and more costly to compensate for the lack of skills late in one's life.

ALMP effectiveness may be moderated not only by individual but also overall workforce education at a state level. The national educational level can shape employers' expectations about jobseekers and constrain the set of strategies that firms can pursue (Estevez‐Abe et al., 2001). When general skills are low overall, companies tend to specialise more in production that uses low and general skills, taking advantage of low wages and flexibility to lay off workers. In such contexts, firms may be more inclined to participate in ALMPs to gain access to cheap short‐term labour (Martin, 2004, p. 56), negatively affecting employment probability after ALMP participation. In countries that invest more in general education and skills acquisition, firms have higher skill needs and are more likely to make longer‐term skill investments (Iversen & Stephens, 2008; Martin & Swank, 2012). Thus, ALMP may be more effective at increasing participants' employment chances in more highly educated societies. Since employers' skill needs and set of strategies are likely to be shaped over long periods of time, changes in national‐level educational achievement can be expected to take years to produce any notable changes in this relationship.

### 3.2 Substitution and compensation

Another plausible education‐ALMP interaction dynamic is that of substitution, with employment chances being less sensitive to ALMP efforts when workforce education is high. Due to rapid technological and structural change, economies whose policies favour general education are expected to have better employment outcomes (Hanushek et al., 2017). Individuals with general education are more able to operate new production technologies, are faster to adopt new technologies, and are more likely to receive career‐related training (Krueger & Kumar, 2004). Having good general skills, such as literacy, mathematics, and information technology knowledge, is a precondition for acquiring more technical skills (Iversen & Stephens, 2008, p. 609). The combination of general and specific skills, in turn, is sought for by firms and therefore increases individuals' employment chances.

Following this argument, higher educational investments may have a substitutionary effect whereby peoples' employment chances are rendered less dependent on ALMP provision as jobseekers are able to find jobs more easily and for longer time spans. When prior educational investments have been low, in turn, ALMPs can have a compensatory role. ALMPs should have a positive access bias as they commonly target disadvantaged unemployed people by design (Bonoli & Liechti, 2018). This entails higher ALMP effectiveness at lower educational levels when individuals' own educational attainment is considered.

### 3.3 Analytical framework

**FIGURE 1:**
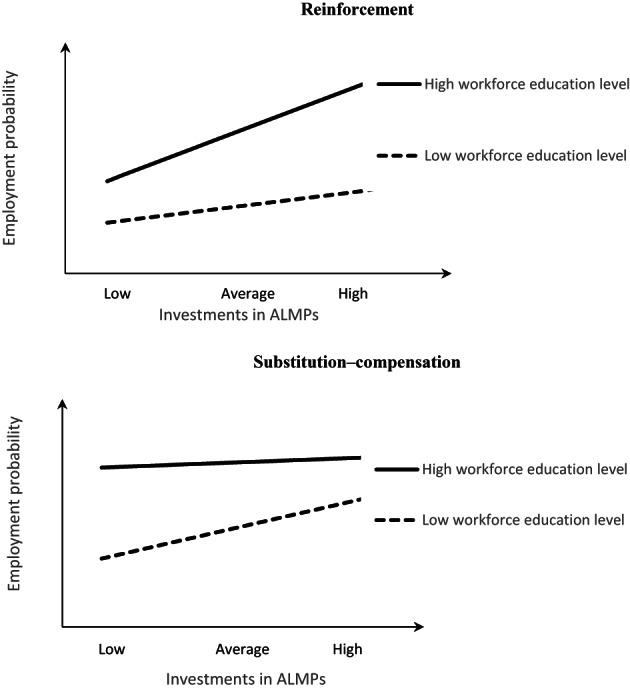
Schematic presentation of education‐ALMP interaction effects on individual employment outcomes. ALMP, active labour market policy. *Source*: Author's illustration

Building on these theoretical considerations, we can conjecture two alternative hypotheses of policy complementarity, schematically depicted in Figure 1. Under the reinforcement hypothesis (top panel), education and ALMPs are mutually reinforcing, the former multiplying the positive effect of investing in the latter to promote employment. This is illustrated with a steeper association between ALMP investments and employment probability when prior investments in education are high. This may apply to educational investments both at an individual and national level. Under the substitution–compensation hypothesis (bottom panel), educational investments substitute for the need for ALMPs to achieve a similar employment target, and their lack thereof can be compensated by higher ALMP efforts. Under this scenario, we can expect a weaker association between ALMP efforts and employment when workforce educational level is high, and stronger ALMP effectiveness when prior educational investments have been low. This is expected to apply particularly when considering individuals' own education.

### 3.4 Heterogeneity in policy complementarities

It is important to acknowledge that the education‐ALMP interplay may not follow the same dynamic for all the working‐age population. Due to their higher risk of being excluded from the labour market increased attention is focused on youth and older workers (see, for instance, European Commission, 2021). Schooling structures that emphasise general education tend to increase employment probability among the youth (Hanushek et al., 2017). When young jobseekers lack job experience but have the necessary general skills to build on, ALMP investments targeted at this group may be more effective at increasing their chances to compete for jobs. Thus, we can expect education and ALMP efforts to be mutually reinforcing especially for this group. Older workers are also a target group of ALMPs, although additional preventive measures such as lifelong education and continuous training may be needed to increase their employability (Walker, 2002, p. 128). Potential differences across age groups will be studied separately in the empirical part of this paper. Another potential source of heterogeneity is programme diversity in ALMPs which may interact differently with education. It has been found, for instance, that in some contexts, the highly educated tend to benefit more from ALMP‐training programmes and the lower educated from ALMP‐employment programmes (e.g. in Sweden by Nordlund, 2011). It is therefore important to disentangle total ALMP efforts when studying how they interact with education.

## 4 METHODOLOGY

### 4.1 Data

The empirical part of this paper assesses the education‐ALMP interaction among the working‐age population in Europe. Analyses draw on micro‐level data from the European Union Statistics on Income and Living Conditions (EU‐SILC) longitudinal surveys 2003–2015 from 29 European countries, retrieved from the statistical office of the European Union (Eurostat).^1^ The longitudinal EU‐SILC data were merged using the *eusilcpanel* tool developed by Borst (2018), and combined with aggregate country‐year data retrieved from Eurostat and OECD databases. Table 1 lists the countries and surveys covered. The analytical sample comprises adults between age 20 and 64 followed for 2 to 4 years. The first survey year observation is excluded from models due to the inclusion of a lagged dependent variable (described under research strategy). The final panel sample with valid data on relevant micro and macro variables comprises 1,551,653 observations from 285 country‐year surveys.

**TABLE 1:** Analysed countries and survey years

Country	Years	Country	Years	Country	Years
Austria	2004–2015	Greece	2006–2015	Poland	2005–2015
Belgium	2004–2015	Hungary	2005–2015	Portugal	2004–2015
Bulgaria	2006–2015	Ireland	2004–2015	Romania	2007–2015
Croatia	2011–2015	Italy	2004–2015	Slovakia	2005–2015
Cyprus	2005–2015	Latvia	2005–2015	Slovenia	2005–2015
Czech Republic	2005–2015	Lithuania	2005–2015	Spain	2004–2015
Denmark	2003–2015	Luxembourg	2003–2015	Sweden	2004–2015
Estonia	2004–2015	Malta	2006–2015	Switzerland	2011–2015
Finland	2004–2015	Netherlands	2005–2015	United Kingdom	2005–2011
France	2004–2015	Norway	2003–2015		

### 4.2 Variables

The dependent variable is individual self‐defined employment status, operationalised as a binary measure equal to 1 if an individual at the time of interview was employed, and 0 if not employed. The category ‘employed’ comprises full‐time and part‐time employees and self‐employed; ‘not employed’ comprises the unemployed and inactive persons, including those fulfilling domestic tasks and care. Analyses exclude pupils, students, the permanently disabled or unfit to work, those in retirement and those in compulsory community or military service. In this final sample, 80% of observations were categorised as ‘employed’ and 20% as ‘not employed’. Among those not employed, 20% switched status to employed during the observed 2‐ to 4‐year period.

The first independent variable of interest is active labour market policy (ALMP) effort. It is operationalised as public spending on ALMPs as a percentage of GDP, divided by the share of unemployed. Data were retrieved from Eurostat's Labour Market Policy expenditure summary tables (Eurostat, 2022a), in combination with the social protection spending database from the OECD (2022) for missing cases.^2^ The ALMP spending measure comprises public employment services, training, employment incentives, supported employment and rehabilitation, direct job creation, and start‐up incentives (categories 1–2 and 4–7). Auxiliary analyses are performed excluding training measures (category 2) from total ALMP spending, narrowing ALMP efforts to those directly related to job matching and job creation. This is done to account for possible heterogeneity in how education relates to ALMP‐employment versus ALMP‐training programmes, and to prevent the risk that the ALMP measure and the adult learning measure (used as a control variable, described below) capture the same phenomenon. Figure S1 shows correlation between the two measures. To ensure that social spending ‘effort’ is not driven by fluctuations in the number of beneficiaries (Ronchi, 2018), ALMP spending measures are adjusted by target population, dividing spending by unemployment in percentage of population in the labour force aged from 20 to 64 years retrieved from Eurostat.^3^ In the analysed country‐year sample, average adjusted ALMP spending was 0.08 (between 0.004 in Greece in 2012 and 0.39 in Denmark in 2008), and 0.06 when excluding training programmes.

The second independent variable—educational investments—is captured using individual and macro‐level indicators of workforce educational attainment. It is important to note that the selected indicators capture not only public investments but also individual effort. No comparative data on public spending on education are available for the period when the analysed sample was of school age. Moreover, in the European context, not only school costs and quality, but also regulation of school admission selection linked to tracking and differentiation are important factors determining school transitions (Van de Werfhorst & Mijs, 2010). Other important factors such as opportunity costs and family background also influence educational opportunities (Breen et al., 2009). Thus, current workforce educational attainment is considered here as a proxy for past investments in general education broadly defined.

Individual‐level education is defined as respondent's highest educational level attained according to the International Standard Classification of Education (ISCED). For the base models, it is coded into three categories: *low educational attainment* comprising no diploma, incomplete or complete primary education (24.9% of the sample); *average education* including lower or higher secondary or post‐secondary but not higher education (46.6%); and *higher education* including college and university (28.5%). Within the *low educational attainment* category, previous literature suggests variation in ALMP effectiveness, with the least disadvantaged among this risk group potentially benefitting more from ALMPs (Bonoli & Liechti, 2018, p. 897). Thus, additional analyses with a more detailed breakdown are performed, distinguishing between individuals with no or incomplete (9%) and complete primary education (16% of the sample).

National‐level education is measured as a share of persons aged from 25 to 64 years with tertiary education (levels 5–8 according to the ISCED 2011 classification) retrieved from Eurostat (2022c ). The selected cut‐off identifies the proportion of *highly educated workforce*, which in the analysed country‐year sample ranges from 12.3% in Italy in 2005 to 45.9% in Luxembourg in 2014.

**FIGURE 2:**
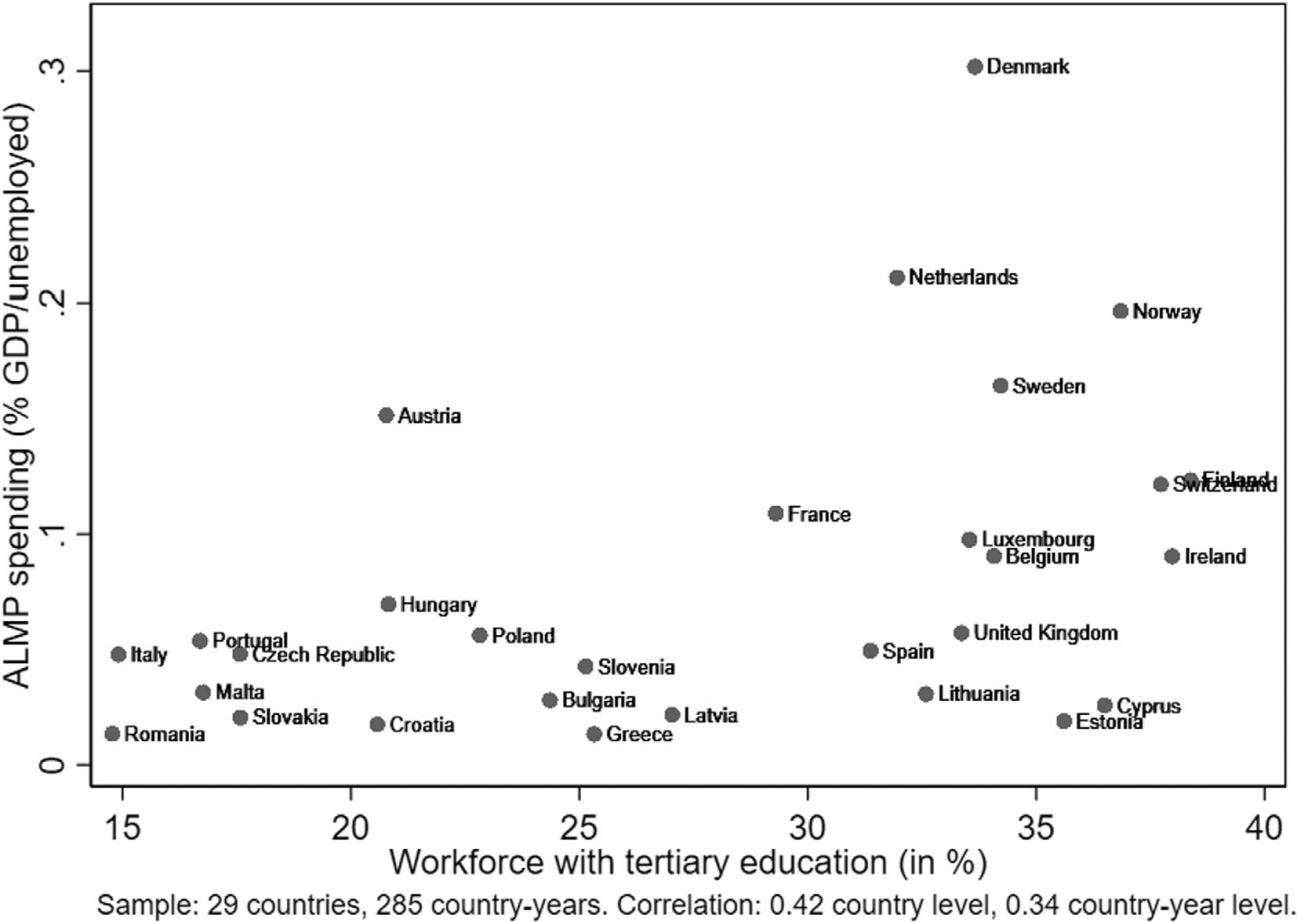
Total ALMP spending efforts and share of highly educated workforce, country averages 2004–2015

Figure 2 visualises how the analysed countries fare in terms of national‐level workforce education and ALMP efforts. Several country groupings emerge, such as those with a low level of both (e.g., Italy, Malta, Romania); average‐high education but low ALMP (the Baltic states, Cyprus, Spain, the United Kingdom); average level of both (e.g., Austria, France); average ALMP and high education (e.g., Finland, Ireland and Switzerland); and high level of both (Denmark, Netherlands, Norway, Sweden). Note that the figure presents country averages while annual values vary between 2004 and 2015, changing country position depending on the observation year. Country groupings are therefore indicative and do not reflect sample distribution. Figure S2 shows annual data plotting country‐years.

*Adult learning and training* programme participation constitutes an important source of continuous knowledge acquisition that can increase individuals' competitiveness and employability. Contrary to ALMPs, adult learning and training are also available to those with stable employment and tends to be taken up by better educated individuals (Bills & van de Werfhorst, 2018). Countries that invest in education and ALMPs also tend to invest in adult learning. In the analysed country‐year sample, adult learning participation rates correlate strongly with national workforce education (corr = 0.54) and ALMP efforts (corr = 0.74; supplement, Figures S3–S4). Thus, the education‐ALMP ‘effect’ on employment may be spurious, driven by lifelong learning. To eliminate the risk of this confounder, base models include a control for adult learning, expressed as participation rates in adult education and training (in last 4 weeks) among population aged from 25 to 64 years retrieved from Eurostat (2022b), with a range from 1% in Bulgaria to over 30% in Denmark and Switzerland.

All models control for a set of covariates to cancel out factors that may influence individuals' employment status at the time of interview. At the individual level, these are: *past employment status* in the previous survey year; *age* at the interview; *gender*; *self‐reported general health* ranging between 1 (very good) and 5 (very bad); *marital status* equal to 1 if married and 0 if otherwise; *household size*; and number of *very small children* under age 2. At the macro‐level, selected controls account for national macro‐economic conditions and the overall welfare state generosity that are known to influence individuals' employment chances. These are: purchasing power adjusted *GDP* per capita at current market prices (Eurostat, 2022d); *unemployment* expressed as a percentage of population in the labour force aged from 20 to 64 years (Eurostat, 2022f); and *total annual social protection expenditure* as percentage of GDP after subtracting spending on old age since the analyses focus on working‐age adults (Eurostat, 2022e). All indicators and descriptive statistics are listed in Table S1, supplementary material.

### 4.3 Research strategy

Analyses use logistic random‐effects dynamic panel multilevel regression models to account for the binary nature of the dependent variable and the three‐level structure of panel data – observations nested in households and country‐years. The random effects are allowed to covary with random intercepts. While it is advisable to nest also within countries when fitting multilevel models to comparative longitudinal survey data (Schmidt‐Catran & Fairbrother, 2016), including this level faced problems of convergence. To account for possible bias from disregarding repeated observations on countries as nested within countries and to cancel out time‐invariant country‐level confounders, baseline models were re‐estimated with country fixed effects as an alternative model specification. Estimates are compared in the findings section and full models are reported in supplementary material.

All models include a lagged dependent variable – respondents' employment status in previous survey year. The inclusion of lagged dependent variables is a common strategy to increase estimate accuracy reducing the threat of omitted variable bias (Shumway & Stoffer, 2017). Two separate analyses are performed to estimate the education‐ALMP interplay: a cross‐level interaction between individual‐level educational attainment and country‐year‐level ALMP effort; and a country‐year‐level interaction between workforce educational attainment and ALMP effort, both measured at a country‐year level. Estimates are expressed in odds ratios, with the interaction term below 1 indicating substitution, equal to 1 indicating no moderation by workforce education, and above 1 pointing at reinforcement. Interaction terms from logit models are shown also in predicted probabilities. Models are run for the total workforce and by age group distinguishing between youth, prime working age and older workers, with age cut‐offs 20–29, 30–54, and 55–64, respectively.

### 4.4 Sensitivity analyses

A range of sensitivity checks were performed to see if results are sensitive to alternative model specifications, operationalization decisions and sample selection. First, as already anticipated, models were re‐estimated with country fixed effects. Second, results were weighted for the inverse of sample size of each country‐year to correct for sample size differences across analysed surveys. Third, models were run without a lagged dependent variable and without controls. Fourth, in the case of the country‐year‐level education‐ALMP interaction, models were re‐estimated with control for individual educational attainment to see how national workforce education moderates ALMP net of one's own educational attainment. Next, as outlined in the variable section, supplementary analyses were performed to check for sensitivity to variable operationalization decisions; ALMP training measures (category 2) were excluded from the total ALMP spending measure; the *low educational attainment* category was re‐defined to differentiate between the most and the least disadvantaged among the *low attainment* group; the ALMP spending measure was lagged by 1 year. In addition, to check for sensitivity to sample selection, country‐years without Eurostat data on ALMP were excluded, and analyses were replicated removing observations from one country at a time and from certain country‐groups.

## 5 FINDINGS

In this section, we empirically test the education‐ALMP interplay in affecting employment chances in the European context. Table 2 presents estimated cross‐level interaction effects of individuals' educational attainment and country‐year‐level aggregate ALMP efforts, controlling for individual‐ and macro‐level covariates and previous year's employment status to avoid selection bias into employment. Table 3 presents the same for country‐year‐level interaction of aggregate educational attainment and ALMP efforts. Estimates are expressed in odds ratios. In both tables, Model 1 shows the direct effects of education and ALMP net of covariates, Model 2 adds a control for adult learning participation, and Model 3 adds an education‐ALMP interaction. Models 4–6 show the interaction by age‐group. Empty models without controls are reported in Tables S3 and S9, and aggregate bivariate associations are plotted in Figure S5, online supplement.

**TABLE 2:** Odds ratios of being employed: Interaction between ALMP spending efforts and individual‐level workforce educational attainment

	Total workforce			Age		
				20–29	30–54	55–64
	M1	M2	M3	M4	M5	M6
Educational attainment level						
Low: no/primary (omitted)						
Average: secondary	1.605***	1.604***	1.603***	1.771***	1.540***	1.474***
	[0.013]	[0.013]	[0.014]	[0.036]	[0.017]	[0.033]
High: college/university	2.575***	2.573***	2.551***	3.112***	2.303***	2.512***
	[0.024]	[0.024]	[0.025]	[0.079]	[0.029]	[0.071]
ALMP efforts (standardised)	1.281***	1.204***	1.221***	1.151***	1.260***	1.316***
	[0.035]	[0.036]	[0.038]	[0.051]	[0.042]	[0.070]
Interaction education‐ALMP						
Average: secondary			0.996	1.017	0.984	0.955
			[0.012]	[0.030]	[0.015]	[0.027]
High: college/university			0.957***	1.108***	0.919***	0.921**
			[0.013]	[0.039]	[0.016]	[0.030]
Adult learning participation (standardised)		1.131***	1.132***	1.051	1.122***	1.193***
		[0.030]	[0.030]	[0.034]	[0.031]	[0.052]
Employed in previous year	69.414***	69.378***	69.355***	25.041***	71.224***	237.087***
	[0.870]	[0.869]	[0.869]	[0.749]	[1.416]	[19.447]
Individual‐level controls	Yes	Yes	Yes	Yes	Yes	Yes
National‐level controls	Yes	Yes	Yes	Yes	Yes	Yes
Observations	1,551,653	1,551,653	1,551,653	227,137	1,058,114	266,402
Households	971,300	971,300	971,300	187,330	738,291	230,019
Country‐years	285	285	285	285	285	285
Variance country‐years	0.078	0.072	0.072	0.081	0.075	0.176
Variance households	0.354	0.354	0.354	0.653	0.326	0.561
Log‐likelihood	−389,383	−389,373	−389,365	−80,212	−247,700	−54,325

*Note*: Estimates from mixed‐effects logistic random intercept dynamic panel regression models. Standard errors in brackets. Significance: ***p < 0.01, **p < 0.05, *p < 0.1. Full models in Table S2, online supplement.

*Source*: Author's calculations using Eurostat EU‐SILC longitudinal data 2003–2015.

**TABLE 3:** Odds ratios of being employed: Interaction between ALMP spending efforts and national workforce educational attainment

	Total workforce			Age		
				20–29	30–54	55–64
	M1	M2	M3	M4	M5	M6
Highly educated workforce, % (standardised)	1.101***	1.052**	1.089***	1.061**	1.099***	1.143***
	[0.025]	[0.025]	[0.028]	[0.028]	[0.029]	[0.049]
ALMP efforts (standardised)	1.316***	1.234***	1.198***	1.183***	1.212***	1.248***
	[0.041]	[0.041]	[0.040]	[0.045]	[0.043]	[0.070]
Interaction Highly educated‐ALMP			1.107***	1.075**	1.108***	1.165***
			[0.032]	[0.033]	[0.034]	[0.057]
Adult learning participation (standardised)		1.151***	1.124***	1.024	1.105***	1.142**
		[0.035]	[0.037]	[0.034]	[0.036]	[0.059]
Employed in previous year	77.694***	77.658***	77.656***	28.579***	78.082***	252.837***
	[0.988]	[0.987]	[0.987]	[0.881]	[1.587]	[21.218]
Individual‐level controls	Yes	Yes	Yes	Yes	Yes	Yes
National‐level controls	Yes	Yes	Yes	Yes	Yes	Yes
Observations	1,551,653	1,551,653	1,551,653	227,137	1,058,114	266,402
Households	971,300	971,300	971,300	187,330	738,291	230,019
Country‐years	285	285	285	285	285	285
Variance country‐years	0.094	0.087	0.083	0.071	0.087	0.209
Variance households	0.379	0.379	0.379	0.738	0.342	0.511
Log‐likelihood	−394,593	−394,583	−394,577	−81,529	−250,401	−54,950

*Note*: Estimates from mixed‐effects logistic random intercept dynamic panel regression models. Standard errors in brackets. Significance: ***p < 0.01, **p < 0.05, *p < 0.1. Full models in Table S8, supplement.

Source: Author's calculations using Eurostat EU‐SILC longitudinal data 2003–2015.

*Cross‐level interaction: individual educational attainment and national ALMP efforts*

Corroborating what is generally found in the literature, estimates in Table 2 show that educational attainment and ALMP efforts are both positively associated with employment. Compared to individuals with no or only primary education, the odds of being employed are 60% greater for those with secondary education, and more than two‐and‐a‐half times greater for the highly‐educated, net of previous employment status and other covariates (M1). Likewise, one standard deviation higher, national ALMP efforts increase individuals' odds to be employed by around 28%. As expected, some of the estimated ALMP effect is captured by adult learning participation (M2). Importantly for this analysis, the interaction effect is negative implying substitution (M3). All else equal, the positive ALMP effect on the odds of being employed is weaker for those with higher compared to lower education (1.221 + (0.957–1) = 1.178, *p*‐value < 0.01). This implies diminishing marginal returns to higher ALMP efforts for the highly educated, and increased ALMP effectiveness for the lower‐educated.

**FIGURE 3:**
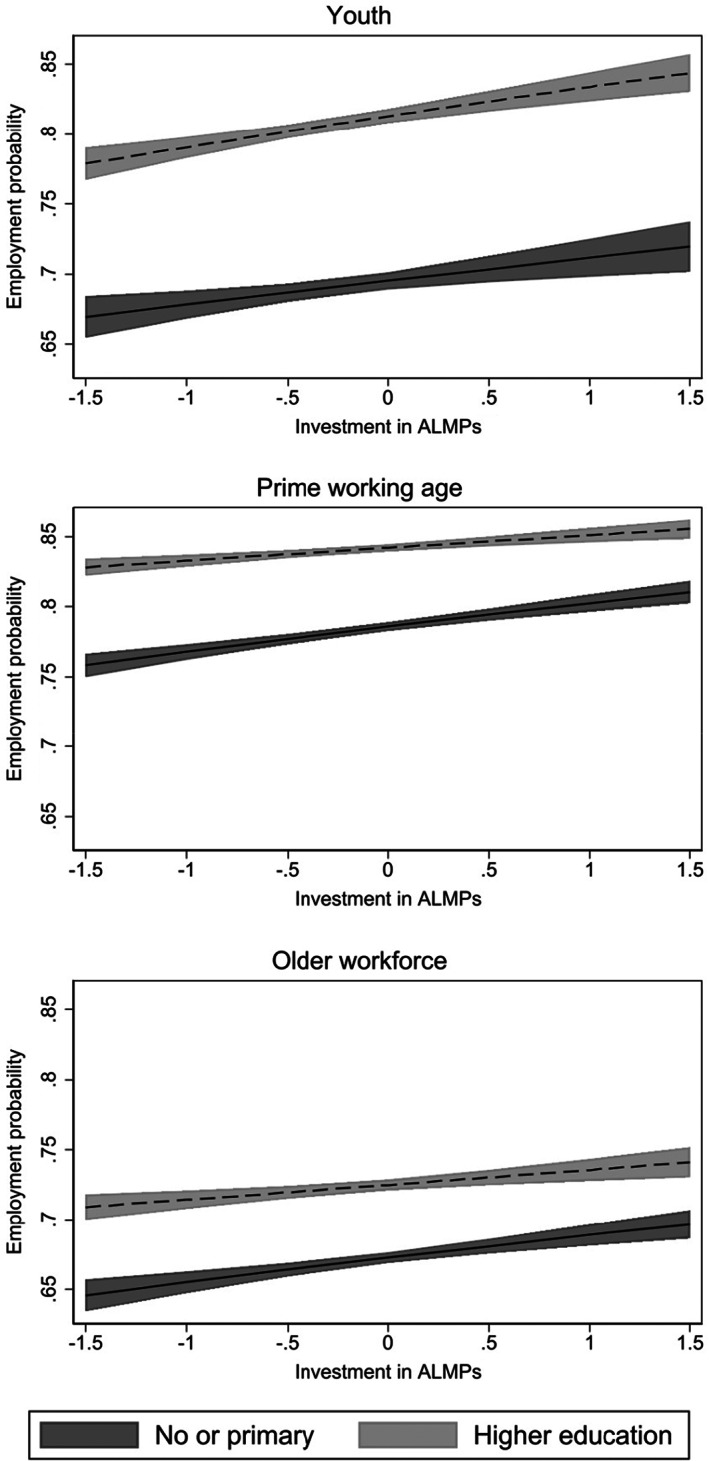
Predicted employment probability at different ALMP effort levels: Interaction with individual level education

Further analyses by age (M4–M6) reveal that the estimated averages for the total workforce do not apply equally to all. To have a clearer understanding of the interaction effects, estimates are expressed in predicted employment probabilities and plotted across the ALMP spending distribution in Figure 3. The X‐axis captures ALMP spending distribution ranging between −1.5 and 1.5 standard deviations to match the sample distribution and exclude extreme values (most country‐years have below‐average spending centred around −1 standard deviation, see Figures 2 and S2). The centre of the *X*‐axis represents contexts with average ALMP spending efforts. The lighter (dashed) and darker (solid) lines represent sampled individuals with higher and lower educational attainment, respectively.

A clear substitution‐compensation pattern in the education‐ALMP interplay is observed for people of prime working age and older workforce (middle and bottom panels, Figure 3). When educational attainment is low, predicted employment probability increases with higher ALMP spending efforts, with an estimated increase of around 5 percentage points across the ALMP distribution. To give a scale of these predicted probabilities, consider that total employment rates among resident population aged from 20 to 64 years across the analysed country‐years ranges from 53% in Greece and Romania to 82% in Switzerland. When educational attainment is high, the association between ALMP efforts and employment chances is weaker, with predicted employment probability increasing by around 3 percentage points across the ALMP spending range. The identified diminishing marginal returns do not seem to be driven by a ceiling effect since employment probability is far from being saturated, especially for older workforces.

For youth, the education‐ALMP interaction dynamic goes in the opposite direction implying reinforcement (top panel, Figure 3). Higher ALMP efforts are associated with higher employment chances among young people of all educational attainment levels, but this positive association is stronger for highly educated youth. The gap in employment probability between low‐ and highly‐educated youth increases from 11 to 13 percentage points across the ALMP distribution. Auxiliary analyses show that the reinforcement effect for this group is larger when considering only ALMP measures directly related to employment, such as job matching, job insertion or direct job creation (Table S4, supplement).

The estimated interaction effects are almost identical when adding country fixed effects as an alternative model specification (Table S5, supplement). The estimates are larger in magnitude and with increased statistical certainty when accounting for sample differences across analysed surveys. Estimates are not sensitive to other performed model specifications, operationalization decisions and sample selection (Tables S5–S7, supplement). In line with theoretical expectations, substitution effect is stronger when considering the least disadvantaged among the *lower educated* group – those with completed primary education (Table S6, supplement). The latter sensitivity test indicates that ALMP effect on employment is lower for the most disadvantaged – those with practically no prior formal education.

*Country‐year‐level interaction: national educational attainment and ALMP efforts*

Table 3 shows estimates for national‐level education‐ALMP interaction effects on individuals' employment chances. Similar to the analysis above, higher national educational attainment and ALMP effort are associated with increased odds to be employed (M1), with some of this effect explained through adult learning participation (M2). Importantly, unlike individual education, national educational attainment moderates ALMP efforts in a reinforcing way (M3). A higher share of highly educated workforce is associated with higher ALMP effectiveness (1.198 + (1.107–1) = 1.305, *p*‐value < 0.01).

**FIGURE 4:**
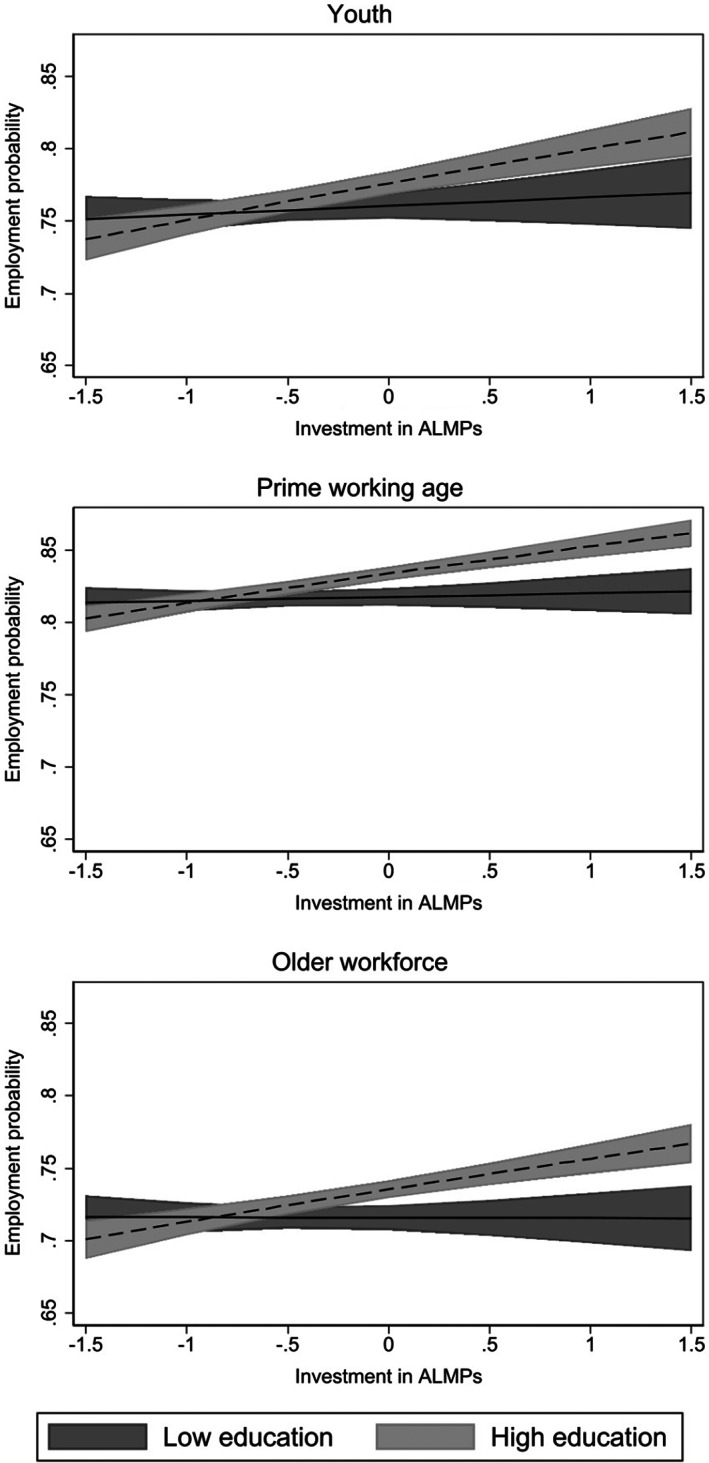
Predicted employment probability at different ALMP effort levels: Interaction with national level education

Figure 4 illustrates this by age group expressed in predicted probabilities based on M4–M6 estimates. The lighter (dashed) and darker (solid) lines represent contexts with high and low shares of workforce with tertiary education measured at the 90th and 10th percentiles of the distribution. Estimates show that employment probability varies little by ALMP effort in contexts where a low share of workforce has tertiary education, but is positively related to ALMP in contexts with a high share of highly educated workforce. High national workforce education seems to increase ALMP effectiveness in promoting employment especially for an older workforce, and less so for youth. The latter finding might be due to a higher pool of competition in highly educated contexts where lack of job experience may be a more prominent obstacle for job (re)insertion.

The estimated reinforcement effect remains close to identical when controlling for individual educational attainment, and when excluding the training category from the total ALMP measure (Tables S10 and S11, supplement). Results are not altered by most other model specifications and sample selection choices (Tables S12 and S13, supplement). One notable exception is the inclusion of country fixed effects, after which the reinforcement effect disappears. The observation period is relatively brief (3–10 years), which may explain the null effects when introducing country fixed effects. As acknowledged in the theoretical framework, within‐country change in the national general skills level may have a lagged moderating effect on ALMP effectiveness since it may take longer time periods to alter work‐level strategies and hiring practices.

Estimates presented here do not imply causality since other unobserved welfare state characteristics beyond education and ALMPs may drive the observed patterns. All models control for the macroeconomic context, welfare state generosity and adult learning participation, but other factors may not be accounted for here.

## 6 CONCLUSIONS AND DISCUSSION

This paper theorised and empirically assessed education‐ALMP complementarities affecting employment chances among a large sample of working‐age individuals residing in 29 European countries. Two alternative hypotheses were proposed: reinforcement predicting higher ALMP effectiveness when workforce education is high; and substitution‐compensation predicting diminishing ALMP returns at higher education levels and increased ALMP effectiveness at lower education levels. Education‐ALMP interplay was tested considering individual and national education separately as this distinction has not only theoretical but also policy‐relevant implications.

When considering individual‐level education, results are in line with the substitution–compensation hypothesis. A highly educated workforce tends to be less dependent on ALMP efforts: their employment chances are relatively higher and vary less across the ALMP distribution. For a lower‐educated workforce, employment probability tends to increase at higher levels of ALMP effort, implying that activation policies somewhat compensate for the lack of prior educational investment. The identified substitution‐compensation effect is reassuring since it means that ALMPs benefit lower‐educated individuals more, contrary to the Matthew Effects assumption. Nevertheless, even with high ALMP efforts, employment probability among lower educated individuals is lower than among a highly educated workforce, indicating that the employment gap between low and highly educated does not close even at high ALMP levels. For youth, education and ALMPs are mutually reinforcing, with higher ALMP effectiveness for highly educated youth. This indicates that lower educated youth is at increased risk of labour market exclusion and benefits less from ALMPs, implying that more targeted interventions could be considered.

When considering national workforce education and how it relates to ALMP efforts, results reveal a clear pattern of reinforcement. Investments in ALMPs, combined with a large share of highly educated workforce, are associated with a substantially higher employment probability compared to the same ALMP efforts but a lower share of highly educated workforce. This applies to all age groups, with somewhat weaker reinforcement among youth. This might be because in settings with high workforce education, it is harder to activate youth, also at high ALMP spending levels.

Regarding policy implications, this paper shows that national workforce educational attainment has bearings on ALMP effectiveness, and therefore the choice of optimal labour market policy strategies. Relative abundance of a highly educated workforce constitutes a reinforcing effect whereby employment transitions can be boosted by more ALMP efforts. The identified reinforcement dynamic implies that high employment levels can be reached when coupling investments in formal education with ALMP. Low educational investments are associated with a lower employment probability and less effective ALMPs which are unable to compensate low general education levels.

The findings of substitution for individual‐level education and reinforcement for macro‐level educational attainment may seem contradictory but are theoretically reconcilable. The substitution effect when individual education is considered indicates that ALMPs are more beneficial and/or more likely to reach the lower educated. As outlined in the theoretical framework, this may be driven by increased employability and self‐sufficiency among highly educated jobseekers and/or a positive ALMP access bias among the lower educated jobseekers, or other mechanisms not described here. The reinforcement effect when considering macro‐level education, in turn, underlines that ALMP effectiveness depends on the overall workforce education, with a more highly educated workforce at state level boosting ALMP effectiveness in promoting employment. Theoretically, it was argued that this may stem from differential work‐level production strategies and subsequent hiring behaviour, but it could also result from better‐quality ALMP service provision or other welfare state characteristics not accounted for here. An important future research avenue is to test the theorised micro‐mechanisms behind the identified interaction effects to uncover the causes of these associations. It is also important to capture longer time periods so that within‐country variation could be exploited better.

## Supporting information


**DATA S1.** Supporting information.

## Data Availability

Data sharing is not applicable to this article as no new data were created or analyzed in this study.

## Endnotes

^1^
The responsibility for all conclusions drawn from the data lies entirely with the author.

^2^
OECD data were used for 5 out of 285 country‐years: Switzerland 2012–2015 and the UK 2011. For country‐years with available data in both databases, the correlation of the ALMP spending measure is 0.99. When excluding country‐years for which Eurostat data are not available, results are highly similar (see Supplemental material).

^3^
The largest share of ALMPs in Europe is targeted at unemployed persons (European Commission, 2021).
