# Supplementary material for: Education and active labour market policy complementarities in promoting employment: Reinforcement, substitution and compensation
Source: Soc Policy Adm. 2023 Jan 10;57(2):235–53. doi: 10.1111/spol.12894 (PMC10108074; doi:10.1111/spol.12894)
Supplement: Supplementary file 1 — DATA S1. Supporting information. [file SPOL-57-235-s001.pdf]

Education and active labour market policy complementarities in promoting employment:
Reinforcement, substitution and compensation

Ilze Plavgo

Supplementary material

Table S1. Descriptive statistics of all micro and macro level variables before transformation

	Obs.	Mean	Std. dev.	Min	Max
**Individual-level indicators**					
Economic status: employed (ref. unemployed)	2,660,852	0.797			
Economic status in previous year*(t-1)*: employed	1,837,013	0.797			
Age at the date of interview	3,244,762	43.11	12.64	20	64
Female (ref. male)	3,244,679	0.514			
Self-reported health on a scale from good to bad	2,841,752	2.130	0.885	1	5
Marital status: Married (ref. single/divorced/separated)	3,235,302	0.598			
Educational level (highest ISCED level attained):	3,214,961				
No diploma		0.005			
Primary education not completed successfully		0.085			
Primary education		0.159			
Lower/higher/post-secondary, not higher education		0.469			
Higher education (college)		0.037			
Higher education (university or HBO5/BES)		0.245			
Household size	3,238,765	3.290	1.428	1	28
Number of children under age 2 in household	3,244,762	0.068	0.281	0	6
Age cohorts:	3,244,762				
20–29 (youth)		0.284			
30–54 (prime working age)		0.383			
55–64 (older workforce)		0.333			
**Macro-level indicators**					
ALMP spending on categories 1-7, in % of GDP (adjusted for % unemployed): Eurostat & OECD	285	0.084	0.075	0.004	0.391
Eurostat only	280	0.084	0.076	0.004	0.391
ALMP spending, excluding spending on training	285	0.062	0.058	0.002	0.319
Population from 25 to 64 years with tertiary education	285	27.88	8.38	12.30	45.90
Participation rate in adult education and training (last 4 weeks), among population aged from 25 to 64 years	285	11.03	7.96	1.30	32.70
Unemployment as share of labour force, age 20–64	285	8.54	4.57	2.00	27.30
GDP current prices (PPS) per capita	285	26735	12006	9964	77564
Social protection expenditure, excluding old age	285	14.17	3.96	6.20	22.30

Sources: Individual-level variables from EU-SILC longitudinal data; aggregate data from Eurostat and OECD (see main text for sources).

Figure S1. Correlation between total ALMP spending and ALMP spending without training, country averages 2004–2015 (top panel) and by country-year (bottom panel)

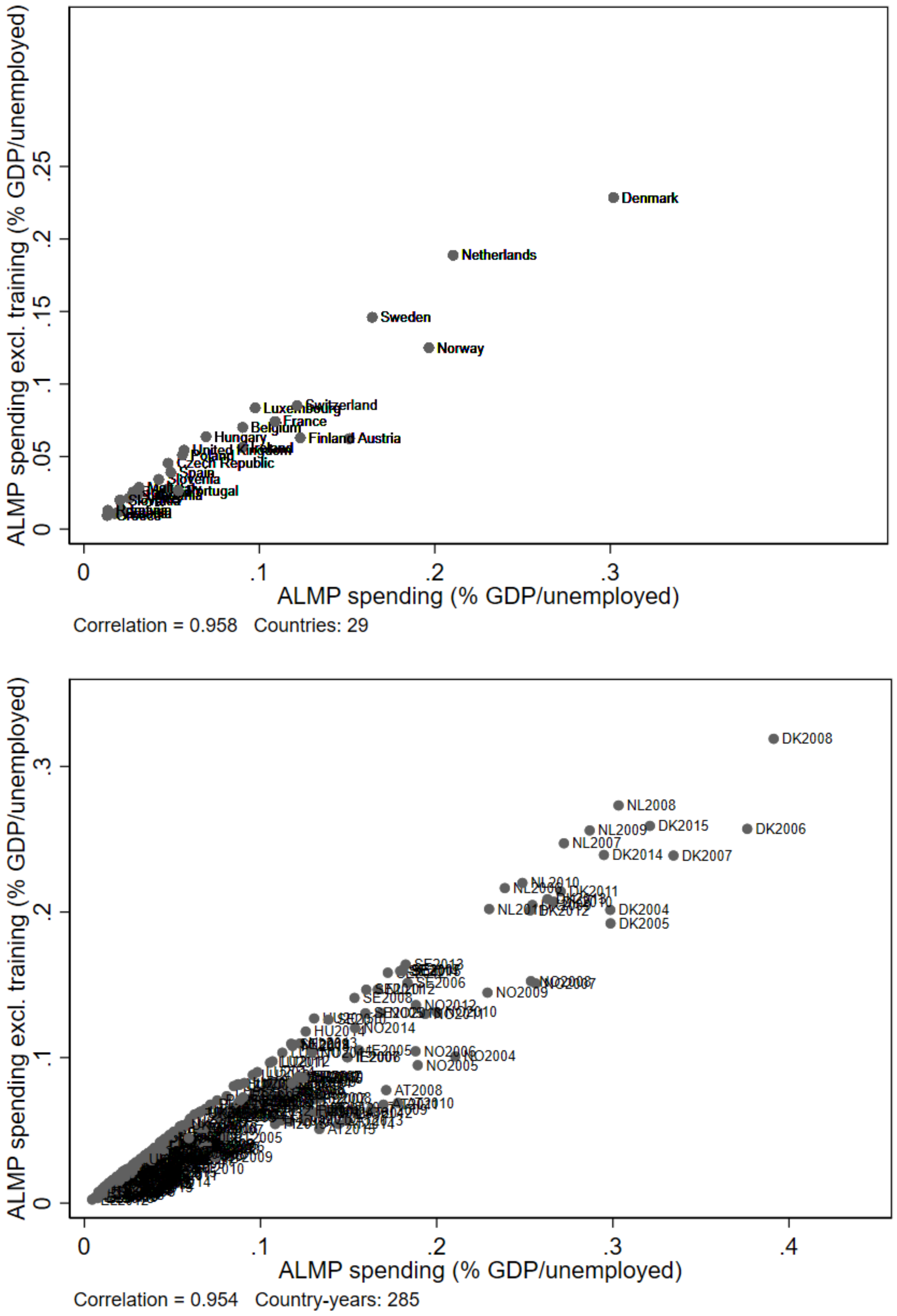

Figure S2. ALMP spending and share of highly educated workforce, for each country-year

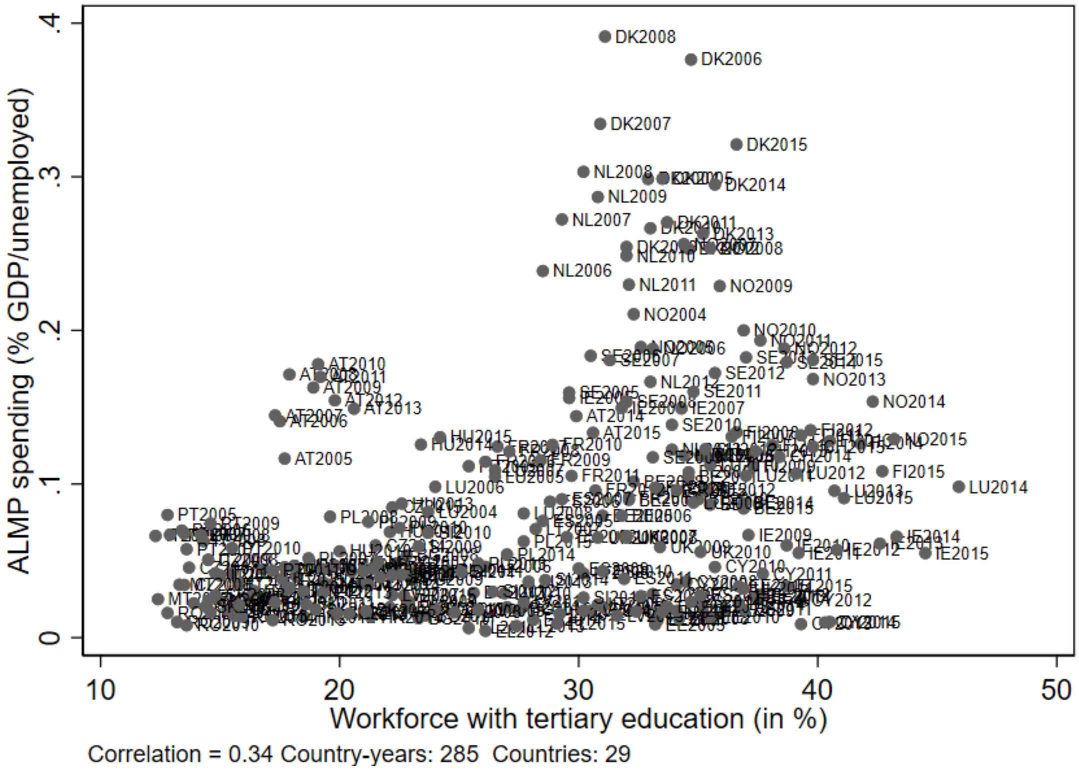

Figure S3. Share of highly educated workforce and participation in adult learning (averages, 2004–2015)

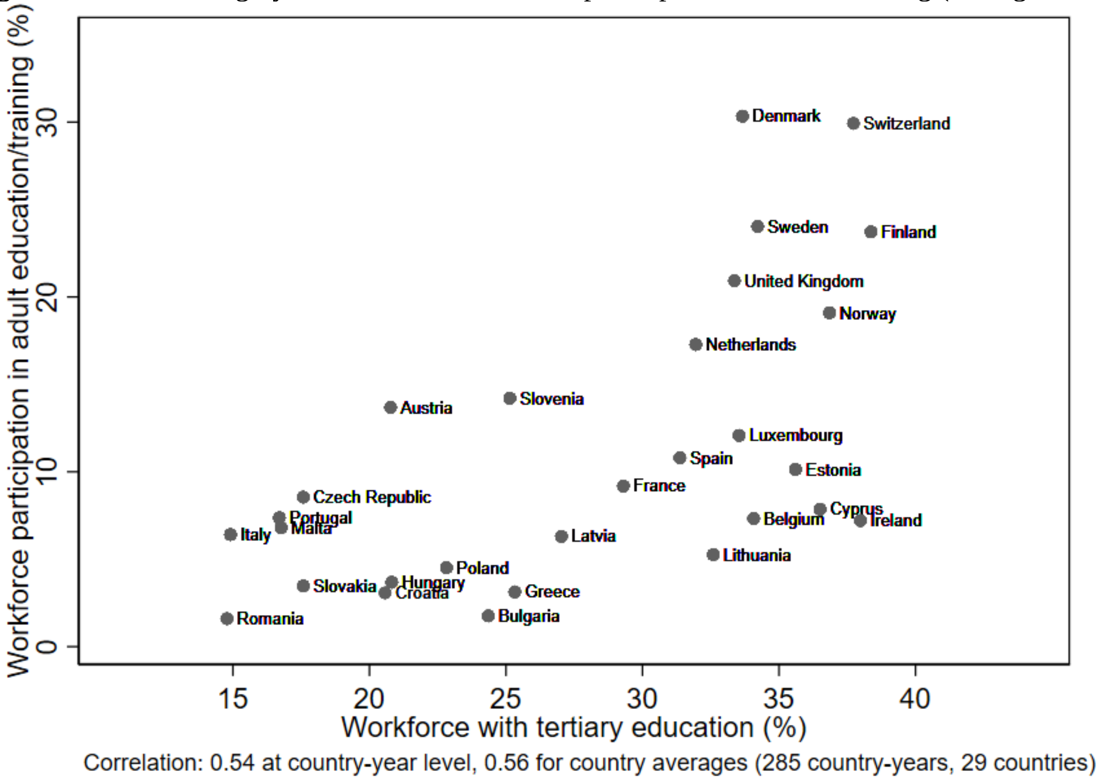

Figure S4. ALMP spending and participation in adult learning (country averages, 2004–2015)

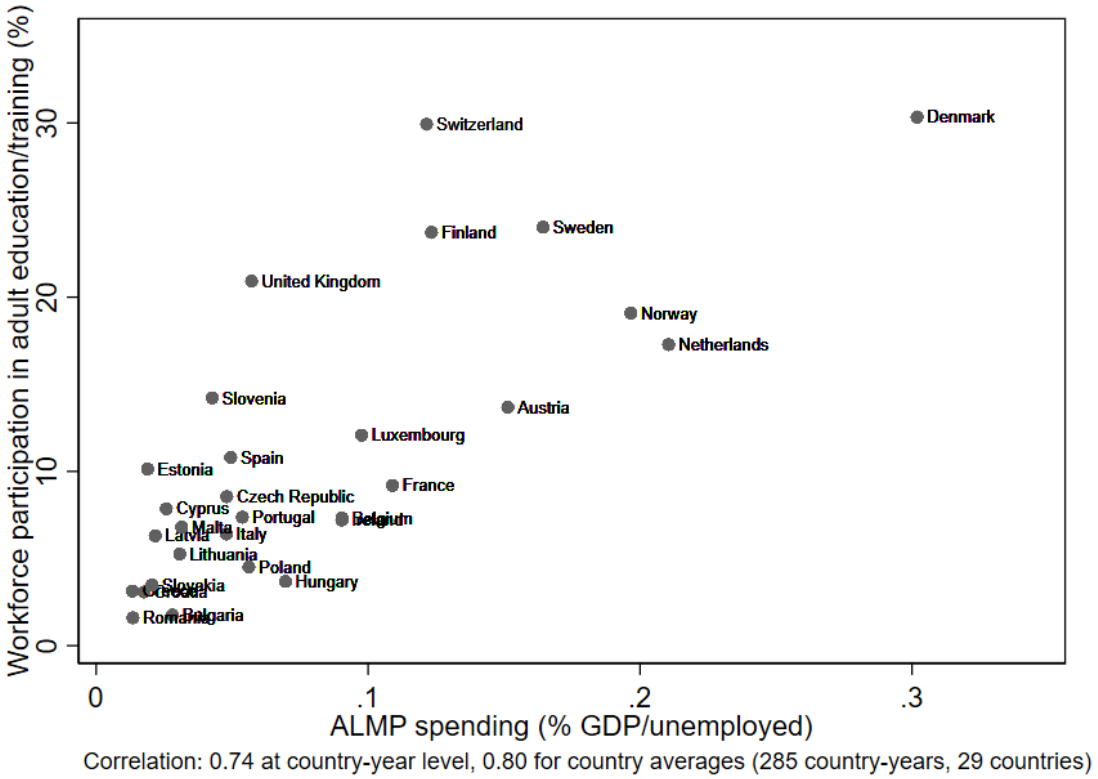

Figure S5. Association between ALMP efforts, workforce education and employment rates (country averages, 2004–2015)

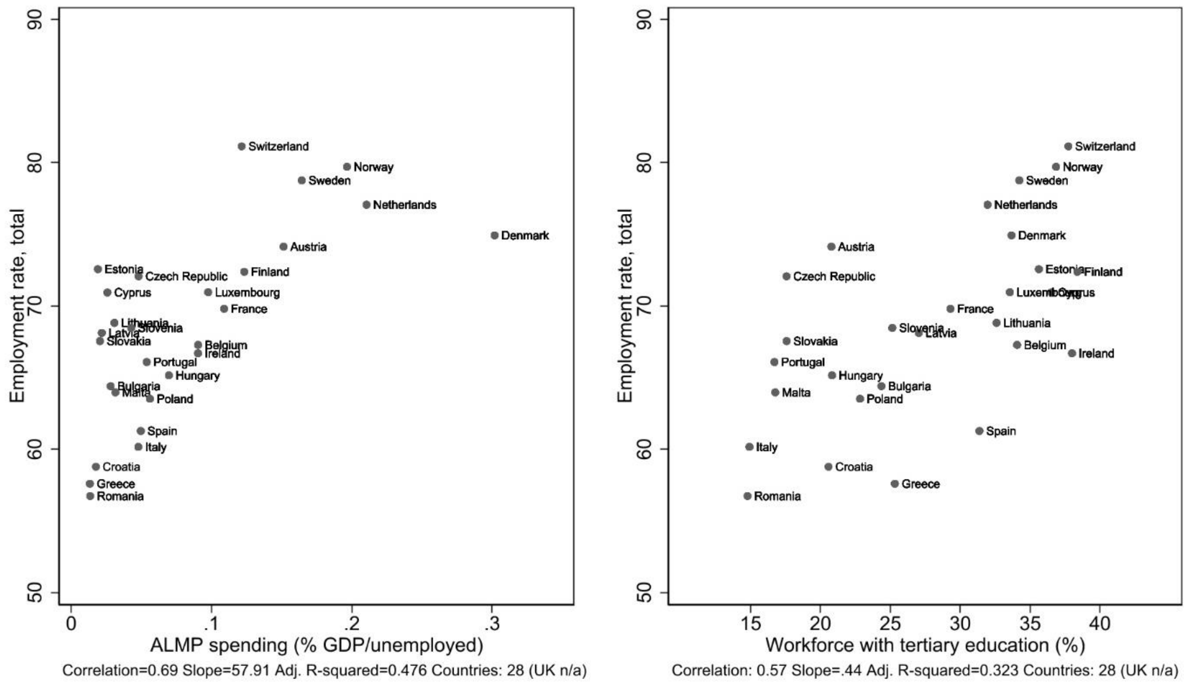

Table S2. Odds ratios of being employed: interaction between ALMP spending efforts and individual-level workforce educational attainment

	Total workforce			Age 20–29	30–54	55–64
	M1	M2	M3	M4	M5	M6
Educational attainment level						
Low: no/primary (omitted)						
Average: secondary	1.605***	1.604***	1.603***	1.771***	1.540***	1.474***
	[0.013]	[0.013]	[0.014]	[0.036]	[0.017]	[0.033]
High: college/university	2.575***	2.573***	2.551***	3.112***	2.303***	2.512***
	[0.024]	[0.024]	[0.025]	[0.079]	[0.029]	[0.071]
ALMP efforts (standardized)	1.281***	1.204***	1.221***	1.151***	1.260***	1.316***
	[0.035]	[0.036]	[0.038]	[0.051]	[0.042]	[0.070]
Interaction Education-ALMP:						
Average: secondary			0.996	1.017	0.984	0.955
			[0.012]	[0.030]	[0.015]	[0.027]
High: college/university			0.957***	1.108***	0.919***	0.921**
			[0.013]	[0.039]	[0.016]	[0.030]
Adult learning participation (standardized)		1.131***	1.132***	1.051	1.122***	1.193***
		[0.030]	[0.030]	[0.034]	[0.031]	[0.052]
Employed in previous year	69.414***	69.378***	69.355***	25.041***	71.224***	237.087***
	[0.870]	[0.869]	[0.869]	[0.749]	[1.416]	[19.447]
Age	0.995***	0.995***	0.995***			
	[0.000]	[0.000]	[0.000]			
Gender: female (*ref. male*)	0.507***	0.507***	0.506***	0.552***	0.494***	0.542***
	[0.003]	[0.003]	[0.003]	[0.009]	[0.004]	[0.010]
Self-reported health (good to bad)	0.772***	0.772***	0.772***	0.831***	0.752***	0.744***
	[0.003]	[0.003]	[0.003]	[0.009]	[0.004]	[0.009]
Married (*ref. not married/separate*)	1.127***	1.127***	1.127***	0.876***	1.171***	0.936***
	[0.008]	[0.008]	[0.008]	[0.016]	[0.011]	[0.021]
Household size	0.978***	0.978***	0.978***	0.921***	0.956***	1.025***
	[0.002]	[0.002]	[0.002]	[0.005]	[0.003]	[0.009]
Number of children under age 2	0.515***	0.515***	0.515***	0.490***	0.548***	0.889*
	[0.005]	[0.005]	[0.005]	[0.010]	[0.007]	[0.061]
Unemployment rate	0.963***	0.964***	0.964***	0.946***	0.963***	0.976***
	[0.004]	[0.004]	[0.004]	[0.005]	[0.004]	[0.007]
GDP per capita	1.000**	1.000***	1.000***	1.000***	1.000***	1.000***
	[0.000]	[0.000]	[0.000]	[0.000]	[0.000]	[0.000]
Social protection spending	0.972***	0.966***	0.966***	0.970***	0.972***	0.932***
	[0.006]	[0.006]	[0.006]	[0.007]	[0.006]	[0.010]
Observations	1,551,653	1,551,653	1,551,653	227,137	1,058,114	266,402
Households	971,300	971,300	971,300	187,330	738,291	230,019
Country-years	285	285	285	285	285	285
Variance country-years	.078	.072	.072	.081	.075	.176
Variance households	.354	.354	.354	.653	.326	.561
Log-likelihood	-389383	-389373	-389365	-80212	-247700	-54325

Source: Author's calculations using Eurostat EU-SILC longitudinal data 2003–2015.
Note: Estimates from mixed-effects logistic random intercept dynamic panel regression models.
Standard errors in brackets. Significance: *** p<0.01, ** p<0.05, * p<0.1.

Table S3. Odds ratios of being employed: interaction between ALMP spending efforts and individual-level workforce educational attainment, empty models

	Base model (total workforce)	Without controls	Without controls with interaction	Without lagged dependent variable
Educational attainment level				
Low: no/primary (omitted)				
Average: secondary	1.603***	2.398***	2.389***	2.340***
	[0.014]	[0.013]	[0.014]	[0.015]
High: college/university	2.551***	4.632***	4.572***	4.992***
	[0.025]	[0.030]	[0.031]	[0.039]
ALMP efforts (standardized)	1.221***	1.498***	1.526***	1.299***
	[0.038]	[0.039]	[0.041]	[0.056]
Interaction Education-ALMP:				
Average: secondary	0.996		0.987	0.984*
	[0.012]		[0.008]	[0.009]
High: college/university	0.957***		0.950***	0.897***
	[0.013]		[0.009]	[0.009]
Adult learning participation (standardized)	1.132***			1.294***
	[0.030]			[0.048]
Employed in previous year	69.355***			
	[0.869]			
Age	0.995***			1.003***
	[0.000]			[0.000]
Gender: female (*ref. male*)	0.506***			0.273***
	[0.003]			[0.001]
Self-reported health (good to bad)	0.772***			0.709***
	[0.003]			[0.002]
Married (*ref. not/separated*)	1.127***			1.197***
	[0.008]			[0.007]
Household size	0.978***			0.913***
	[0.002]			[0.002]
Number of children under age 2	0.515***			0.586***
	[0.005]			[0.005]
Unemployment rate	0.964***			0.952***
	[0.004]			[0.006]
GDP per capita	1.000***			1.000***
	[0.000]			[0.000]
Social protection spending	0.966***			0.969***
	[0.006]			[0.009]
Observations	1,551,653			1,551,653
Households	971,300			971,300
Country-years	285			285
Variance country-years	.070	.186	.187	.016
Variance households	.354	.142	.142	.453
Log-likelihood	-389365	-720667		-671778

Source: Author's calculations using Eurostat EU-SILC longitudinal data 2003–2015.
Notes: Estimates from mixed-effects logistic random intercept dynamic panel regression models.
Significance: *** p<0.01, ** p<0.05, * p<0.1. Standard errors in brackets.

Table S4. Odds ratios of being employed: interaction between ALMP spending efforts and individual-level workforce educational attainment: ALMP measure comprising employment-related programmes excluding training (category 2)

	Total workforce	Age 20–29	Age 30–54	Age 55–64	Total, with country fixed effects
Educational attainment level					
Low: no/primary (omitted)					
Average: secondary education	1.606***	1.774***	1.543***	1.476***	1.603***
	[0.014]	[0.037]	[0.017]	[0.034]	[0.014]
High: college/university	2.557***	3.154***	2.304***	2.513***	2.555***
	[0.025]	[0.082]	[0.029]	[0.071]	[0.025]
ALMP efforts (standardized)	1.202***	1.195***	1.255***	1.243***	1.054
	[0.033]	[0.049]	[0.037]	[0.059]	[0.036]
Interaction Education-ALMP:					
Average: secondary education	1.005	1.024	0.992	0.971	1.006
	[0.012]	[0.032]	[0.016]	[0.026]	[0.012]
High: college/university	0.972**	1.153***	0.932***	0.933**	0.971**
	[0.013]	[0.043]	[0.016]	[0.029]	[0.012]
Adult learning participation rate (standardized)	1.134***	1.038	1.120***	1.213***	0.99
	[0.029]	[0.032]	[0.030]	[0.053]	[0.043]
Employed in previous year	69.372***	25.005***	71.249***	236.656***	69.222***
	[0.869]	[0.748]	[1.416]	[19.407]	[0.867]
Age	0.995***				0.995***
	[0.000]				[0.000]
Gender: female (*ref. male*)	0.506***	0.552***	0.494***	0.543***	0.506***
	[0.003]	[0.009]	[0.004]	[0.010]	[0.003]
Self-reported health (good to bad)	0.772***	0.832***	0.752***	0.744***	0.771***
	[0.003]	[0.009]	[0.004]	[0.009]	[0.003]
Married (*ref. not/separated*)	1.127***	0.875***	1.171***	0.937***	1.128***
	[0.008]	[0.016]	[0.011]	[0.021]	[0.008]
Household size	0.978***	0.921***	0.956***	1.025***	0.978***
	[0.002]	[0.005]	[0.003]	[0.009]	[0.002]
Number of children under age 2	0.515***	0.491***	0.548***	0.888*	0.515***
	[0.005]	[0.010]	[0.007]	[0.061]	[0.005]
Unemployment rate	0.962***	0.948***	0.962***	0.972***	0.966***
	[0.004]	[0.005]	[0.004]	[0.007]	[0.003]
GDP per capita	1.000***	1.000***	1.000***	1.000***	1.000**
	[0.000]	[0.000]	[0.000]	[0.000]	[0.000]
Social protection spending	0.970***	0.970***	0.975***	0.939***	0.933***
	[0.006]	[0.006]	[0.006]	[0.009]	[0.010]
Observations	1,551,653	227,137	1,058,114	266,402	1,551,653
Households	971,300	187,330	738,291	230,019	971,300
Country-years	285	285	285	285	285
Variance country-years	.070	.075	.071	.179	.019
Variance households	.354	.651	.326	.558	.355
Log-likelihood	-389365	-80197	-247697	-54328	-389204

Table S5. Odds ratios of being employed: interaction between ALMP spending efforts and individual-level workforce educational attainment, alternative model specifications

	Base model (total workforce)	Country fixed effects	Weight for inverse of sample size	Lagged ALMP (1 year)
Educational attainment level				
Low: no/primary (omitted)				
Average: secondary	1.603***	1.600***	1.675***	1.603***
	[0.014]	[0.014]	[0.037]	[0.014]
High: college/university	2.551***	2.544***	2.623***	2.565***
	[0.025]	[0.026]	[0.060]	[0.026]
ALMP efforts (standardized)	1.221***	1.04	1.226***	1.199***
	[0.038]	[0.039]	[0.046]	[0.041]
Interaction Education-ALMP:				
Average: secondary	0.996	0.998	0.961*	1.003
	[0.012]	[0.012]	[0.022]	[0.012]
High: college/university	0.957***	0.957***	0.947**	0.973**
	[0.013]	[0.013]	[0.024]	[0.013]
Adult learning participation (standardized)	1.132***	0.995	1.124***	1.141***
	[0.030]	[0.041]	[0.026]	[0.033]
Employed in previous year	69.355***	69.214***	69.709***	69.698***
	[0.869]	[0.867]	[2.798]	[0.919]
Age	0.995***	0.995***	0.994***	0.996***
	[0.000]	[0.000]	[0.001]	[0.000]
Gender: female (*ref. male*)	0.506***	0.506***	0.522***	0.518***
	[0.003]	[0.003]	[0.013]	[0.004]
Self-reported health (good to bad)	0.772***	0.771***	0.788***	0.767***
	[0.003]	[0.003]	[0.007]	[0.004]
Married (*ref. not/separated*)	1.127***	1.128***	1.101***	1.130***
	[0.008]	[0.008]	[0.015]	[0.009]
Household size	0.978***	0.978***	0.994	0.979***
	[0.002]	[0.002]	[0.005]	[0.003]
Number of children under age 2	0.515***	0.515***	0.491***	0.514***
	[0.005]	[0.005]	[0.022]	[0.006]
Unemployment rate	0.964***	0.965***	0.964***	0.961***
	[0.004]	[0.004]	[0.004]	[0.005]
GDP per capita	1.000***	1.000**	1.000***	1.000**
	[0.000]	[0.000]	[0.000]	[0.000]
Social protection spending	0.966***	0.932***	0.971***	0.965***
	[0.006]	[0.010]	[0.005]	[0.007]
Observations	1,551,653	1,551,653	1,551,653	1,398,340
Households	971,300	971,300	971,300	875,559
Country-years	285	285	285	256
Variance country-years	.070	.019	.000	.076
Variance households	.354	.355	.386	.360
Log-likelihood	-389365	-389202		-351412
Log-pseudolikelihood			-68.56	

Source: Author’s calculations using Eurostat EU-SILC longitudinal data 2003–2015.
Notes: Estimates from mixed-effects logistic random intercept dynamic panel regression models.
Significance: *** p<0.01, ** p<0.05, * p<0.1. Standard errors in brackets.

Table S6. Odds ratios of being employed: interaction between ALMP spending efforts and individual-level workforce educational attainment, more detailed operationalization of educational attainment

	More detailed break-down within the *lower educated* category
Educational attainment level	
Low: primary (omitted)	
Lowest: no or incomplete primary	0.798***
	[0.011]
Average: secondary	1.491***
	[0.014]
High: college/university	2.370***
	[0.026]
ALMP spending efforts (standardized)	1.229***
	[0.039]
Interaction Education-ALMP:	
Lowest: no or incomplete primary	0.952**
	[0.021]
Low: primary (omitted)	1
	[0.000]
Average: secondary	0.989
	[0.013]
High: college/university	0.950***
	[0.013]
Adult learning participation (standardized)	1.116***
	[0.029]
Employed in previous year	69.313***
	[0.869]
Age	0.996***
	[0.000]
Gender: female (*ref. male*)	0.506***
	[0.003]
Self-reported health (good to bad)	0.774***
	[0.003]
Married (*ref. not/separated*)	1.126***
	[0.008]
Household size	0.979***
	[0.002]
Number of children under age 2	0.517***
	[0.005]
Unemployment rate	0.964***
	[0.004]
GDP per capita	1.000**
	[0.000]
Social protection spending	0.967***
	[0.006]
Observations	1,551,653
Households	971,300
Country-years	285
Variance country-years	.082
Variance households	.367
Log-likelihood	-390212

Source: Author's calculations using Eurostat EU-SILC longitudinal data 2003–2015.
Significance: *** p<0.01, ** p<0.05, * p<0.1. Standard errors in brackets.

Table S7. Odds ratios of being employed: interaction between ALMP spending efforts and individual-level workforce educational attainment, sensitivity check for sample selection

	Excluding:					
	Countries without Eurostat data	Eastern Europe	Eastern Europe: Age 30-54	Central Europe	Southern Europe	Northern Europe
Educational attainment level						
Low: no/primary (omitted)						
Average: secondary	1.605***	1.581***	1.541***	1.551***	1.650***	1.609***
	[0.014]	[0.015]	[0.018]	[0.015]	[0.017]	[0.015]
High: college/university	2.545***	2.451***	2.228***	2.492***	2.658***	2.545***
	[0.026]	[0.026]	[0.030]	[0.027]	[0.032]	[0.028]
ALMP efforts (standardized)	1.219***	1.223***	1.258***	1.239***	1.269***	1.081**
	[0.039]	[0.041]	[0.046]	[0.041]	[0.041]	[0.040]
Interaction Education-ALMP:						
Average: secondary	0.998	1.009	0.989	1.003	0.942***	1.004
	[0.012]	[0.013]	[0.017]	[0.013]	[0.012]	[0.014]
High: college/university	0.956***	0.987	0.952***	0.963***	0.895***	0.954***
	[0.013]	[0.014]	[0.017]	[0.014]	[0.013]	[0.015]
Adult learning participation (standardized)	1.140***	1.122***	1.115***	1.127***	1.116***	1.081***
	[0.031]	[0.031]	[0.033]	[0.030]	[0.030]	[0.031]
Employed in previous year	69.746***	68.773***	70.439***	71.956***	71.286***	71.524***
	[0.878]	[0.997]	[1.619]	[0.996]	[1.113]	[0.909]
Age	0.995***	0.993***		0.993***	0.995***	0.995***
	[0.000]	[0.000]		[0.000]	[0.000]	[0.000]
Gender: female (*ref. male*)	0.508***	0.488***	0.470***	0.502***	0.527***	0.501***
	[0.003]	[0.004]	[0.005]	[0.004]	[0.004]	[0.003]
Self-reported health (good-bad)	0.771***	0.779***	0.758***	0.778***	0.760***	0.774***
	[0.003]	[0.004]	[0.005]	[0.004]	[0.004]	[0.003]
Married (*ref. not/separated*)	1.130***	1.115***	1.162***	1.131***	1.107***	1.119***
	[0.009]	[0.010]	[0.012]	[0.009]	[0.010]	[0.009]
Household size	0.979***	0.970***	0.944***	0.978***	0.992***	0.974***
	[0.002]	[0.003]	[0.003]	[0.003]	[0.003]	[0.002]
Number of children under age 2	0.515***	0.545***	0.577***	0.612***	0.457***	0.524***
	[0.005]	[0.006]	[0.008]	[0.007]	[0.005]	[0.005]
Unemployment rate	0.964***	0.969***	0.967***	0.962***	0.957***	0.960***
	[0.004]	[0.005]	[0.005]	[0.004]	[0.006]	[0.004]
GDP per capita	1.000**	1.000**	1.000***	1.000***	1.000**	1.000***
	[0.000]	[0.000]	[0.000]	[0.000]	[0.000]	[0.000]
Social protection spending	0.965***	0.966***	0.972***	0.971***	0.967***	0.981***
	[0.006]	[0.008]	[0.008]	[0.006]	[0.006]	[0.006]
Observations	1532122	1219180	835557	1281430	1154065	1439588
Households	958856	783893	592435	803188	748268	859235
Country-years	280	227	227	234	243	239
Variance country-years	.072	.079	.085	.067	.073	.057
Variance households	.354	.293	.285	.333	.358	.360
Log-likelihood	-385030	-302243	-193507	-323374	-270305	-368958

Notes: Countries without Eurostat data: Switzerland 2012–2015 and the UK 2011
Eastern Europe: Estonia, Latvia, Lithuania, Poland, Bulgaria, Romania
Central Europe: Austria, Czeck Republic, Hungary, Slovakia, Slovenia
Southern Europe: Greece, Italy, Portugal, Spain
Northern Europe: Norway, Sweden, Denmark, Finland

Table S8. Odds ratios of being employed: interaction between ALMP spending efforts and national workforce educational attainment

	Total workforce			Age: 20–29	30–54	55–64
	M1	M2	M3	M4	M5	M6
Highly educated workforce, % (standardized)	1.101***	1.052**	1.089***	1.061**	1.099***	1.143***
	[0.025]	[0.025]	[0.028]	[0.028]	[0.029]	[0.049]
ALMP spending efforts (standardized)	1.316***	1.234***	1.198***	1.183***	1.212***	1.248***
	[0.041]	[0.041]	[0.040]	[0.045]	[0.043]	[0.070]
Interaction						
Highly educated workforce-ALMP			1.107***	1.075**	1.108***	1.165***
			[0.032]	[0.033]	[0.034]	[0.057]
Adult learning participation (standardized)		1.151***	1.124***	1.024	1.105***	1.142**
		[0.035]	[0.037]	[0.034]	[0.036]	[0.059]
Employed in previous year	77.694***	77.658***	77.656***	28.579***	78.082***	252.837***
	[0.988]	[0.987]	[0.987]	[0.881]	[1.587]	[21.218]
Age	0.992***	0.991***	0.991***			
	[0.000]	[0.000]	[0.000]			
Gender: female (*ref. male*)	0.534***	0.534***	0.534***	0.622***	0.522***	0.537***
	[0.004]	[0.004]	[0.004]	[0.009]	[0.004]	[0.010]
Self-reported health (good to bad)	0.729***	0.729***	0.729***	0.786***	0.708***	0.702***
	[0.003]	[0.003]	[0.003]	[0.009]	[0.004]	[0.008]
Married (*ref. not married/separated*)	1.152***	1.152***	1.152***	0.857***	1.184***	0.932***
	[0.009]	[0.009]	[0.009]	[0.016]	[0.011]	[0.021]
Household size	0.956***	0.956***	0.956***	0.886***	0.936***	1.017**
	[0.002]	[0.002]	[0.002]	[0.004]	[0.003]	[0.008]
Number of children under age 2	0.532***	0.532***	0.532***	0.489***	0.586***	0.854**
	[0.005]	[0.005]	[0.005]	[0.010]	[0.007]	[0.058]
Unemployment rate	0.961***	0.964***	0.963***	0.949***	0.962***	0.971***
	[0.005]	[0.005]	[0.005]	[0.005]	[0.005]	[0.008]
GDP per capita	1.000***	1.000***	1.000***	1	1.000***	1.000***
	[0.000]	[0.000]	[0.000]	[0.000]	[0.000]	[0.000]
Social protection spending	0.967***	0.961***	0.963***	0.973***	0.969***	0.925***
	[0.007]	[0.007]	[0.007]	[0.007]	[0.007]	[0.010]
Observations	1,551,653	1,551,653	1,551,653	227,137	1,058,114	266,402
Households	971,300	971,300	971,300	187,330	738,291	230,019
Country-years	285	285	285	285	285	285
Variance country-years	.094	.087	.083	.071	.087	.209
Variance households	.379	.379	.379	.738	.342	.511
Log-likelihood	-394593	-394583	-394577	-81529	-250401	-54950

Source: Author’s calculations using Eurostat EU-SILC longitudinal data 2003–2015.

Notes: Estimates from logistic random-effects dynamic panel multilevel regression models. Standard errors [in brackets]. Significance: *** p<0.01, ** p<0.05, * p<0.1

Table S9. Odds ratios of being employed: interaction between ALMP spending efforts and national workforce educational attainment, empty models

	Base model (total workforce)	Without controls	Without controls, with interaction	Without lagged dependent variable
Highly educated workforce, % (standardized)	1.089***	1.078**	1.159***	1.174***
	[0.028]	[0.035]	[0.038]	[0.046]
ALMP spending efforts (standardized)	1.198***	1.539***	1.383***	1.237***
	[0.040]	[0.050]	[0.050]	[0.063]
Interaction				
Highly educated workforce-ALMP	1.107***		1.291***	1.190***
	[0.032]		[0.059]	[0.053]
Adult learning participation (standardized)	1.124***			1.290***
	[0.037]			[0.062]
Employed in previous year	77.656***			
	[0.987]			
Age	0.991***			0.996***
	[0.000]			[0.000]
Gender: female (*ref. male*)	0.534***			0.286***
	[0.004]			[0.002]
Self-reported health (good to bad)	0.729***			0.634***
	[0.003]			[0.002]
Married (*ref. not married/separated*)	1.152***			1.238***
	[0.009]			[0.007]
Household size	0.956***			0.873***
	[0.002]			[0.002]
Number of children under age 2	0.532***			0.611***
	[0.005]			[0.005]
Unemployment rate	0.963***			0.949***
	[0.005]			[0.007]
GDP per capita	1.000***			1.000***
	[0.000]			[0.000]
Social protection spending	0.963***			0.961***
	[0.007]			[0.010]
Observations	1,551,653	1,551,653	1,551,653	1,551,653
Households	971,300	971,300	971,300	971,300
Country-years	285	285	285	285
Variance country-years	.083	.258	.232	.205
Variance households	.379	.351	.351	.662
Log-likelihood/Log-pseudolikelihood	-394577	-753782	-753767	-701258

Table S10. Odds ratios of being employed: interaction between ALMP spending efforts and national workforce educational attainment, with control for individual educational attainment

	Total workforce			Age: 20–29	30–54	55–64
	M1	M2	M3	M4	M5	M6
Highly educated workforce, % (standardized)	1.017	0.978	1.013	1.001	1.019	1.066*
	[0.021]	[0.021]	[0.023]	[0.027]	[0.024]	[0.041]
ALMP efforts (standardized)	1.270***	1.204***	1.171***	1.175***	1.189***	1.219***
	[0.035]	[0.036]	[0.035]	[0.045]	[0.037]	[0.061]
Interaction						
Highly educated workforce-ALMP			1.107***	1.073**	1.107***	1.165***
			[0.029]	[0.034]	[0.030]	[0.051]
Adult learning participation (standardized)		1.131***	1.098***	1.039	1.087***	1.115**
		[0.033]	[0.032]	[0.037]	[0.033]	[0.054]
Employed in previous year	69.114***	69.085***	69.081***	24.973***	70.943***	238.978***
	[0.866]	[0.865]	[0.865]	[0.747]	[1.408]	[19.597]
Age	0.996***	0.996***	0.996***			
	[0.000]	[0.000]	[0.000]			
Gender: female (*ref. male*)	0.507***	0.507***	0.507***	0.549***	0.494***	0.545***
	[0.003]	[0.003]	[0.003]	[0.009]	[0.004]	[0.010]
Self-reported health (good to bad)	0.775***	0.776***	0.776***	0.833***	0.756***	0.749***
	[0.003]	[0.003]	[0.003]	[0.009]	[0.004]	[0.009]
Married (*ref. not married/separated*)	1.124***	1.124***	1.124***	0.876***	1.171***	0.938***
	[0.008]	[0.008]	[0.008]	[0.016]	[0.011]	[0.021]
Education level attained (ref. tertiary):						
No diploma	0.277***	0.278***	0.278***	0.230***	0.324***	0.284***
	[0.011]	[0.012]	[0.012]	[0.027]	[0.018]	[0.025]
Primary education, incomplete	0.323***	0.323***	0.323***	0.285***	0.370***	0.335***
	[0.004]	[0.004]	[0.004]	[0.011]	[0.006]	[0.012]
Primary education, complete	0.395***	0.395***	0.395***	0.318***	0.426***	0.406***
	[0.004]	[0.004]	[0.004]	[0.008]	[0.006]	[0.013]
(Post-)Secondary, not higher	0.591***	0.591***	0.591***	0.545***	0.628***	0.550***
	[0.005]	[0.005]	[0.005]	[0.011]	[0.007]	[0.016]
Higher education (college)	0.723***	0.724***	0.724***	0.692***	0.739***	0.683***
	[0.013]	[0.013]	[0.013]	[0.027]	[0.017]	[0.039]
Household size	0.979***	0.979***	0.979***	0.922***	0.957***	1.026***
	[0.002]	[0.002]	[0.002]	[0.005]	[0.003]	[0.009]
Number of children under age 2	0.515***	0.515***	0.515***	0.491***	0.545***	0.891*
	[0.005]	[0.005]	[0.005]	[0.010]	[0.007]	[0.061]
Unemployment rate	0.963***	0.966***	0.965***	0.947***	0.964***	0.975***
	[0.005]	[0.005]	[0.004]	[0.005]	[0.005]	[0.007]
GDP per capita	1.000**	1.000*	1.000**	1.000***	1.000***	1.000***
	[0.000]	[0.000]	[0.000]	[0.000]	[0.000]	[0.000]
Social protection spending	0.970***	0.965***	0.968***	0.971***	0.973***	0.935***
	[0.006]	[0.006]	[0.006]	[0.007]	[0.006]	[0.009]
Observations	1,551,653	1,551,653	1,551,653	227,137	1,058,114	266,402
Households	971,300	971,300	971,300	187,330	738,291	230,019
Country-years	285	285	285	285	285	285
Variance country-years	.076	.071	.068	.077	.070	.164
Variance households	.358	.357	.357	.655	.326	.577
Log-likelihood	-389069	-389060	-389053	-80162	-247571	-54274

Figure S6. Employment probability at different ALMP effort levels: interaction with national-level education, with control for individual educational attainment

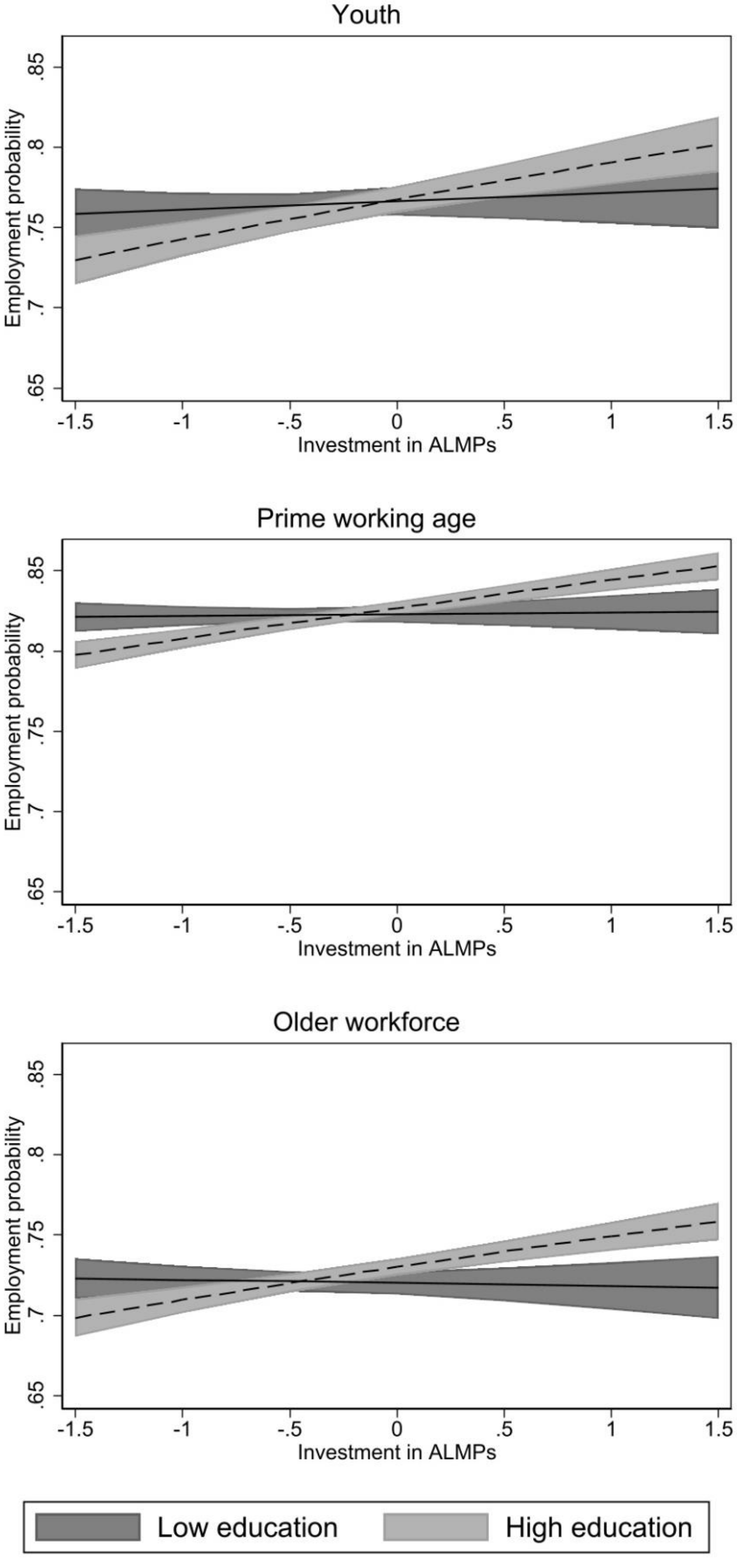

Table S11. Odds ratios of being employed: interaction between ALMP spending efforts and national workforce educational attainment, ALMP measure comprising employment-related programmes excluding training (category 2) (with control for individual educational attainment)

	Total workforce	Age 20–29	Age 30–54	Age 55–64
Highly educated workforce, % (standardized)	1.023	0.991	1.026	1.092**
	[0.027]	[0.031]	[0.028]	[0.049]
ALMP spending efforts (standardized)	1.150***	1.236***	1.181***	1.136***
	[0.033]	[0.045]	[0.035]	[0.054]
Interaction				
Highly educated workforce-ALMP	1.110***	1.045	1.104***	1.188***
	[0.037]	[0.042]	[0.038]	[0.066]
Adult learning participation (standardized)	1.107***	1.039	1.094***	1.138***
	[0.032]	[0.036]	[0.033]	[0.056]
Employed in previous year	69.087***	24.959***	70.971***	238.684***
	[0.865]	[0.746]	[1.409]	[19.567]
Age	0.996***			
	[0.000]			
Gender: female (*ref. male*)	0.507***	0.549***	0.494***	0.545***
	[0.003]	[0.009]	[0.004]	[0.010]
Self-reported health (good to bad)	0.776***	0.833***	0.756***	0.749***
	[0.003]	[0.009]	[0.004]	[0.009]
Married (*ref. not married/separated*)	1.124***	0.875***	1.171***	0.939***
	[0.008]	[0.016]	[0.011]	[0.021]
Education level attained (ref. tertiary):				
No diploma	0.278***	0.230***	0.323***	0.283***
	[0.012]	[0.027]	[0.018]	[0.025]
Primary education, incomplete	0.323***	0.286***	0.369***	0.335***
	[0.004]	[0.011]	[0.006]	[0.012]
Primary education, complete	0.395***	0.318***	0.425***	0.406***
	[0.004]	[0.008]	[0.006]	[0.013]
(Post-)Secondary, not higher	0.591***	0.546***	0.628***	0.551***
	[0.005]	[0.011]	[0.007]	[0.016]
Higher education (college)	0.724***	0.692***	0.739***	0.683***
	[0.013]	[0.027]	[0.017]	[0.039]
Household size	0.979***	0.922***	0.957***	1.025***
	[0.002]	[0.005]	[0.003]	[0.009]
Number of children under age 2	0.515***	0.491***	0.546***	0.892*
	[0.005]	[0.010]	[0.007]	[0.061]
Unemployment rate	0.963***	0.949***	0.962***	0.969***
	[0.004]	[0.005]	[0.004]	[0.007]
GDP per capita	1.000**	1.000***	1.000***	1.000***
	[0.000]	[0.000]	[0.000]	[0.000]
Social protection spending	0.971***	0.970***	0.976***	0.942***
	[0.006]	[0.006]	[0.006]	[0.009]
Observations	1,551,653	227,137	1,058,114	266,402
Households	971,300	187,330	738,291	230,019
Country-years	285	285	285	285
Variance country-years	.067	.072	.068	.168
Variance households	.358	.654	.326	.575
Log-likelihood	-389053	-80154	-247568	-54278

Table S12. Odds ratios of being employed: interaction between ALMP spending efforts and national workforce educational attainment, alternative model specifications

	Base model (total workforce)	Country fixed effects	Weight for inverse of sample size	Lagged ALMP (1 year)
Highly educated workforce, % (standardized)	1.089***	1.031	1.084***	1.090***
	[0.028]	[0.044]	[0.024]	[0.030]
ALMP spending efforts (standardized)	1.198***	1.006	1.203***	1.179***
	[0.040]	[0.037]	[0.043]	[0.044]
Interaction				
Highly educated workforce-ALMP	1.107***	0.975	1.079***	1.116***
	[0.032]	[0.032]	[0.029]	[0.035]
Adult learning participation (standardized)	1.124***	1.004	1.126***	1.130***
	[0.037]	[0.044]	[0.033]	[0.038]
Employed in previous year	77.656***	77.484***	79.033***	78.154***
	[0.987]	[0.985]	[3.344]	[1.045]
Age	0.991***	0.992***	0.990***	0.992***
	[0.000]	[0.000]	[0.001]	[0.000]
Gender: female (*ref. male*)	0.534***	0.534***	0.549***	0.547***
	[0.004]	[0.004]	[0.014]	[0.004]
Self-reported health (good to bad)	0.729***	0.728***	0.747***	0.724***
	[0.003]	[0.003]	[0.007]	[0.003]
Married (*ref. not married/separated*)	1.152***	1.153***	1.122***	1.155***
	[0.009]	[0.009]	[0.016]	[0.009]
Household size	0.956***	0.956***	0.973***	0.956***
	[0.002]	[0.002]	[0.005]	[0.002]
Number of children under age 2	0.532***	0.532***	0.506***	0.530***
	[0.005]	[0.005]	[0.022]	[0.006]
Unemployment rate	0.963***	0.968***	0.965***	0.961***
	[0.005]	[0.004]	[0.004]	[0.005]
GDP per capita	1.000***	1.000**	1.000***	1.000***
	[0.000]	[0.000]	[0.000]	[0.000]
Social protection spending	0.963***	0.931***	0.966***	0.961***
	[0.007]	[0.010]	[0.006]	[0.007]
Observations	1,551,653	1,551,653	1,551,653	1,398,340
Households	971,300	971,300	971,300	875,559
Country-years	285	285	285	256
Variance country-years	.083	.019	.00	.086
Variance households	.379	.381	.414	.387
Log-likelihood	-394577	-394398		-356142
Log-pseudolikelihood			-69.6	

Table S13. Odds ratios of being employed: interaction between ALMP spending efforts and national workforce educational attainment, sensitivity check for sample selection

	Excluding:				
	Countries without Eurostat data	Eastern Europe	Southern Europe	Northern Europe	Northern Europe: Age 30-54 [a]
Highly educated workforce (standardized)	1.089***	1.053*	1.074**	1.054*	1.066**
	[0.028]	[0.032]	[0.033]	[0.028]	[0.031]
ALMP efforts (standardized)	1.194***	1.185***	1.177***	1.112***	1.147***
	[0.041]	[0.043]	[0.042]	[0.045]	[0.050]
Interaction					
Highly educated-ALMP	1.112***	1.142***	1.123***	1.032	1.048
	[0.033]	[0.038]	[0.035]	[0.037]	[0.040]
Adult learning participation (standardized)	1.131***	1.114***	1.116***	1.109***	1.095**
	[0.038]	[0.038]	[0.037]	[0.038]	[0.040]
Employed in previous year	78.132***	76.562***	79.865***	80.248***	81.360***
	[0.998]	[1.126]	[1.264]	[1.035]	[1.676]
Age	0.991***	0.989***	0.992***	0.991***	
	[0.000]	[0.000]	[0.000]	[0.000]	
Gender: female (*ref. male*)	0.536***	0.509***	0.555***	0.528***	0.518***
	[0.004]	[0.004]	[0.004]	[0.004]	[0.005]
Self-reported healh	0.728***	0.736***	0.716***	0.731***	0.709***
	[0.003]	[0.004]	[0.004]	[0.003]	[0.004]
Married	1.154***	1.127***	1.159***	1.142***	1.173***
	[0.009]	[0.010]	[0.010]	[0.009]	[0.011]
Household size	0.956***	0.953***	0.968***	0.951***	0.930***
	[0.002]	[0.003]	[0.003]	[0.002]	[0.003]
No. of children under 2	0.532***	0.560***	0.472***	0.541***	0.598***
	[0.005]	[0.006]	[0.005]	[0.006]	[0.008]
Unemployment rate	0.964***	0.967***	0.967***	0.962***	0.961***
	[0.005]	[0.006]	[0.008]	[0.005]	[0.005]
GDP per capita	1.000***	1.000**	1.000***	1.000***	1.000***
	[0.000]	[0.000]	[0.000]	[0.000]	[0.000]
Social protection spending	0.962***	0.976***	0.966***	0.973***	0.978***
	[0.007]	[0.009]	[0.007]	[0.007]	[0.007]
Observations	1,532,122	1,219,180	1,154,065	1,439,588	984,624
Households	958856	783893	748268	859235	664801
Country-years	280	227	243	239	239
Variance country-years	0.083	0.088	0.090	0.072	.079
Variance households	0.380	0.318	0.382	0.386	.358
Log-likelihood	-390184	-306143	-273934	-373941	-238106

Notes: Countries without Eurostat data: Switzerland 2012–2015 and the UK 2011
Eastern Europe: Estonia, Latvia, Lithuania, Poland, Bulgaria, Romania
Central Europe: Austria, Czeck Republic, Hungary, Slovakia, Slovenia
Southern Europe: Greece, Italy, Portugal, Spain
Northern Europe: Norway, Sweden, Denmark, Finland
[a] Interaction term estimate statistically more precise when controlling for individual level educational attainment.
